# Artificial intelligence empowers full-stack histopathological diagnosis and prognosis of renal cell tumor: a multi-center study with external validation

**DOI:** 10.1186/s12916-026-05110-5

**Published:** 2026-08-04

**Authors:** Ying Xiong, Wei Xi, Gelei Zhang, Xiaoyuan Luo, Li Xiao, Jianbo Gao, Run Wang, Kang Wang, Yun Zhao, Qi Sun, Zilong Wang, Jianming Guo, Le Qu, Yingyong Hou, Di Zhao, Shuo Wang

**Affiliations:** 1 Department of Urology, Zhongshan Hospital, Fudan University, Shanghai, China; 2 Department of Urology, Zhongshan Hospital (Xiamen), Fudan University, Xiamen, China; 3 Department of Control Science and Engineering, Tongji University, Shanghai, China; 4 Digital Medical Research Center, School of Basic Medical Sciences, Fudan University, Shanghai, China; 5 Department of Pathology, Huadong Hospital, Fudan University, Shanghai, China; 6 Department of Urology, The People’s Hospital of Lincang, Lincang, Yunnan China; 7 Department of Pathology, Sir Run Run Shaw Hospital, Hangzhou, China; 8 Department of Pathology, Xiamen Branch, Zhongshan Hospital, Fudan University, Xiamen, China; 9 Microsoft Research Asia, Shanghai, China; 10 Department of Urology, Jinling Hospital, Affiliated Hospital of Medical School, Nanjing University, Nanjing, China; 11 Department of Pathology, Zhongshan Hospital, Fudan University, Shanghai, China; 12 School of Robotics and Automation, Nanjing University, Suzhou, China

**Keywords:** Artificial intelligence, Digital pathology, Renal cell tumor, Diagnosis, Prognosis

## Abstract

**Background:**

The rapid advancement of digital pathology has opened unprecedented opportunities for intelligent diagnosis in renal cell tumor. However, there remains a significant gap in the availability of reliable deep learning models capable of comprehensive kidney cancer detection, classification, grading, and survival prediction.

**Method:**

This study retrospectively analyzed 11,135 whole-slide images (WSIs) from 7033 patients with renal tumor, sourced from four medical centers and two public cohorts. Histopathological representations were extracted using the foundation model Prov-GigaPath. A full-stack renal tumor diagnosis and prognosis framework was developed by combining fully supervised learning and weakly supervised multi-instance learning to enable both regional characterization and patient-level inference.

**Results:**

The deep learning model demonstrated high accuracy in identifying normal tissue (AUC = 0.990), tumor tissue (AUC = 0.982), necrosis tissue (AUC = 0.994), sarcomatoid differentiation (AUC = 0.967), and pseudocapsule tissue (AUC = 0.990) across various pathological types of renal cell tumor. For nine major subtypes of renal cell tumor, classification AUC reached 0.956–0.998 across multi-center validation cohorts. WHO/ISUP nuclear grade prediction for clear cell renal cell carcinoma (ccRCC) and papillary renal cell carcinoma (pRCC) achieved an AUC of 0.867. A whole-slide-derived pan-renal cell tumor pathological risk score independently predicted overall survival and significantly outperformed WHO/ISUP grading in prognostic stratification (*p* < 0.001).

**Conclusions:**

We developed and validated a comprehensive AI framework integrating tissue-region detection, renal tumor subtype classification, nuclear grading, and survival prediction. These findings support its potential as a decision-support tool for renal tumor pathology, while prospective workflow-based studies are warranted to determine its clinical utility and impact on pathologist performance.

**Supplementary Information:**

The online version contains supplementary material available at 10.1186/s12916-026-05110-5.

## Background

Renal cell tumor represents a common urologic malignancy, with an estimated age-standardized rate of 14.5/100,000 [1]. As the 14th most common malignancy globally, renal cell tumor caused 179,368 deaths worldwide in 2020, with a calculated global age-standardized rate of 1.8/100 000 [2]. Pathological assessment has been the golden standard for detection and subtyping of renal cell tumors [3]. However, persistent challenges remained in reporting of renal cell tumor pathology, such as inter-rater disagreement both in subtype classification and tumor nuclear grading, even among experienced pathologist [4]. In 2022 WHO classification of renal tumors, there are now 25 subtypes of renal cell tumors, among which 11 subtypes were molecularly defined, so it becomes increasingly common for there to be difficulty assigning a tumor type. For grading system, although the World Health Organization/International Society of Urological Pathology(WHO/ISUP) grading system is more applicable and reproducible, it is still to a large extent subject to an individual pathologist’s microscopic assessment and sampling of tumors [5]. The grading system only applies to clear cell renal cell carcinoma (ccRCC) and papillary renal cell carcinoma (pRCC). Currently there is no pathological scoring system to take both histologic subtype and tumor grading into account for assessment of patient prognosis, which is desperately needed due to the growing number of tumor subtypes and lack of grading system for other tumor types than ccRCC and pRCC [6, 7]. On the other hand, workload for pathologists increases as a result of more surgeries performed for renal tumors and more complicated diagnosis required in medical treatment, while the shortage of pathologists is worsening [8].

Recent advances in whole-slide imaging (WSI) and artificial intelligence (AI) have brought unprecedented opportunities for clinical practice of renal cell tumor pathology [9]. Applications of AI in digital pathology could reduce interobserver disagreement, improve diagnostic accuracy, and ease the burden of pathologists [10]. More importantly, carrying morphology and spatial arrangement of millions of different cells, whole-slide images contain remarkable information that is beyond interpretation of pathologist and currently not systematically exploited [11]. Deep learning approaches have been developed for tumor detection [12], subtyping [13] and grading [14], and predicting prognosis [15] in plenty of cancer types. These methods could simplify routine works of human pathologists and reduce their workload. For renal cell tumors, two preliminary studies constructed deep learning models for prediction of malignant versus benign regions, classification of three major subtypes of renal cell tumors [ccRCC, pRCC and chromophobe RCC (chRCC)], and survival prediction for ccRCC [16, 17]. The models are trained with cases from public datasets, the Cancer Genome Atlas (TCGA) Kidney Clear Cell Carcinoma (KIRC) cohort, Papillary Cell Carcinoma (KIRP) cohort and Kidney Chromophobe (KICH) cohort. Although there are major limitations in these studies, deep learning on WSIs still demonstrated great potential in smart diagnosis of renal cell tumors.

To address these limitations, we developed and validated a foundation model-enabled, full-stack histopathological AI system that performs automated tissue-level characterization, comprehensive subtype classification, nuclear grade prediction, and WSI-based survival risk modeling across diverse renal cell tumor subtypes, and rigorously evaluated its clinical generalizability in large multi-center cohorts. Our pan-renal cell tumor computational pathology framework establishes a unified histology-to-prognosis paradigm to support precision diagnostics and prognostication in kidney cancer.

## Methods

This was a retrospective, multi-center study aimed at developing and validating a full-stack histopathological artificial intelligence (AI) empowers the comprehensive region detection, subtype classification, nuclear grading, and survival prediction of renal cell tumor.

### Patient cohorts

We retrospectively collected 8303 WSIs of 4809 patients who received partial or radical nephrectomy with available hematoxylin and eosin (H&E) slides and pathologically proved renal cell tumor in Zhongshan Hospital from 2005 to 2022, 222 WSIs of 141 patients in Xiamen Clinical Research Center for Cancer Therapy from 2017 to 2022, 110 WSIs of 110 patients in Zhangye People’s Hospital from 2008 to 2022, and 896 WSIs of 877 patients in Huadong Hospital from 2010 to 2023. Because papillary adenomas are clinically silent and usually discovered as incidental findings [18], this subtype was excluded from further analyses. Patients with bilateral or familial renal cell tumors, mixed histologic subtypes, or a prior history of nephrectomy were also excluded. Detailed information of all patients were listed in Additional file 1: Table 1. For ccRCC, pRCC and chRCC, we collected one or two WSIs for each patient. For other subtypes of renal cell tumors, we collected up to ten WSIs for each patient. Three experienced pathologists in urology (Run Wang, Qi Sun, and Xue Zhang) re-evaluated the slides and pathological reports to re-assign the histologic subtypes, WHO/ISUP grades, and TNM(tumor–node–metastasis) stage of these cases according to the 2022 WHO Classification of Tumors of the Urinary System and Male Genital Organs, the WHO/ISUP grading system, and the 2017 AJCC TNM staging manual, respectively [3, 19]. Cases with suspicious subtypes and insufficient diagnostic information were excluded. The three pathologists also assigned WHO/ISUP grading to non-clear cell or papillary RCC types, which was permitted by international structured reporting protocols, despite that the grading system has not been validated as prognostic parameters for these subtypes [19]. A total of 1604 WSIs of 1096 patients from four public datasets in the Cancer Imaging Archive (TCIA), TCGA-KIRC, TCGA-KIRP, TCGA-KICH, and CPTAC-CCRCC were collected for the TCIA validation set. Basic clinical information and WSIs was obtained from GDC Data Portal (https://portal.gdc.cancer.gov/) [20–27]. The subtypes and gradings of each WSI were re-assessed by the three pathologists based on original WSIs and pathological reports from the Cancer Digital Slide Archive (https://cancer.digitalslidearchive.org/). WSIs with pathologic subtype with clinical information obtained from the GDC cancer portal were excluded.

### WSI preprocessing

To harmonize spatial resolution across WSIs from multiple centers and scanners, all slides were normalized to an approximate resolution of 1 μm per pixel and divided into non-overlapping 224 × 224 patches. Quality control was performed by excluding patches with low color saturation (saturation < 10) or insufficient structural content (edge density ≤ 10). Only regions with reliable pathologist annotations were retained for subsequent analyses. Patch spatial coordinates were preserved for downstream analyses.

### Patch feature encoding

Patch embeddings were extracted using Prov-GigaPath, a Vision Transformer (ViT)-based model pretrained with standard DINOv2 settings on a large-scale dataset comprising 1.3 billion patches. Notably, Prov-GigaPath was pretrained exclusively on a proprietary, real-world dataset from the Providence Health & Services network and does not incorporate any data from public repositories such as TCGA or CPTAC [28]. Accordingly, none of the datasets used in this study—neither our internal multi-center cohorts (Zhongshan, Huadong, Zhangye, and Xiamen), which were independently curated locally and never shared externally, nor the public TCGA/CPTAC cohort, overlapped with the Prov-GigaPath pretraining corpus, thereby precluding any data leakage from the foundation model. Each patch yielded a 1536-dimensional feature vector, producing an *n* × 1536 representation per slide, where n denotes the number of valid tissue patches. These features were subsequently used as inputs for downstream tasks.

### Attention-based multiple instance learning (ABMIL)

To aggregate patch-level features into patient-level predictions, we employed an attention-based multiple instance learning (ABMIL) framework, in which each slide is treated as a bag of patches [29]. The model learns to assign higher attention weights to discriminative regions, thereby focusing on the most informative tissue areas. We adapted the standard ABMIL architecture to enhance feature transformation and attention discrimination. Two different settings were used for the attention module, as detailed in Additional file 1. The final output of the module was a single attention score for each patch, followed by softmax normalization across all instances. These normalized scores were used to compute a weighted aggregation of patch features, which was subsequently passed through a linear classification head for patient-level prediction.

### Task setting

The downstream analysis comprises four consecutive histopathological tasks. First, for tissue component detection, full-slide images were manually annotated by experienced pathologists to identify five distinct micro-regions: normal tissue, tumor tissue, pseudocapsule, necrosis, and sarcomatoid differentiation. The subsequent three tasks were sequentially executed utilizing a refined region-filtering pipeline. To eliminate confounding histological elements, only the benign ‘normal tissue’ patches were excluded, while the remaining four types of patches were collectively preserved as the tumor-associated macro-region. Specifically, the subtype classification network operated exclusively on this comprehensive tumor-associated region to predict the nine major pathological subtypes of renal tumors. For patient-level nuclear grading, tumor-associated patches were aggregated per patient and stratified into low-grade (WHO/ISUP 1–2) and high-grade (WHO/ISUP 3–4) cohorts. Finally, for prognostication, a WSI-based Risk Score (WRS) was computed from the selected tumor-associated regions to predict oncologic outcomes, and its performance was rigorously benchmarked against traditional WHO/ISUP grading and TNM staging systems.

### Model training

For the patch-level classification task, feature representations were processed by a multilayer perceptron (MLP) classification head, which generated probability distributions across five tissue classes. This network consisted of three fully connected layers, each followed by batch normalization and rectified linear unit(ReLU) activation. For pathological type classification, the same architecture was used, with the output layer adjusted to predict nine subtypes. Both networks were trained using stochastic gradient descent (SGD) and optimized with the cross-entropy loss function. For both nuclear grading and prognosis tasks, the ABMIL was employed. Patient level features were constructed by aggregating all patch-level features across patient, which were then fed into the network. The ABMIL model was trained with the AdamW Optimizer, nuclear grading task optimized with the multi-class binary cross-entropy loss and prognosis task optimized with the cross-entropy loss function. For patch-level and slide-level classification, the decision thresholds were selected by maximizing Youden’s J statistic on the training set. These thresholds were applied unchanged to the internal validation and external test cohorts. For other tasks, all cohorts adopt the 0.5 probability threshold. No thresholds were optimized using data or outcome labels from the test cohorts. All hyperparameters and details of each network are all explained in Additional file 1: Table 2.

## Statistical analyses

Statistical analyses were carried out with Python and SPSS(Statistical Package for the Social Sciences) Statistics 21.0. Receiver operating characteristic (ROC) curves were performed to assess diagnostic accuracies of the deep learning models. Recurrence-free survival (RFS) was defined as the time of surgery to the time of local recurrence or distant metastasis. Disease-specific survival (DSS) was calculated from the time of surgery to the time of death from renal cell tumors and overall survival (OS) was calculated from the time of surgery to the time of death from all causes. The survival curves were plotted with Kaplan-Meier analyses and compared by log-rank test. Univariate and multivariable analyses were used to evaluate the prognostic value of pathologic risk score. The prognostic values of WHO/ISUP grading system and pathologic risk score were demonstrated with Harrell’s concordance index (C-index). Statistical tests were two-sided and a p-value < 0.05 was considered statistically significant.

## Results

### Patient recruitment and overview of the study

**Fig. 1:**
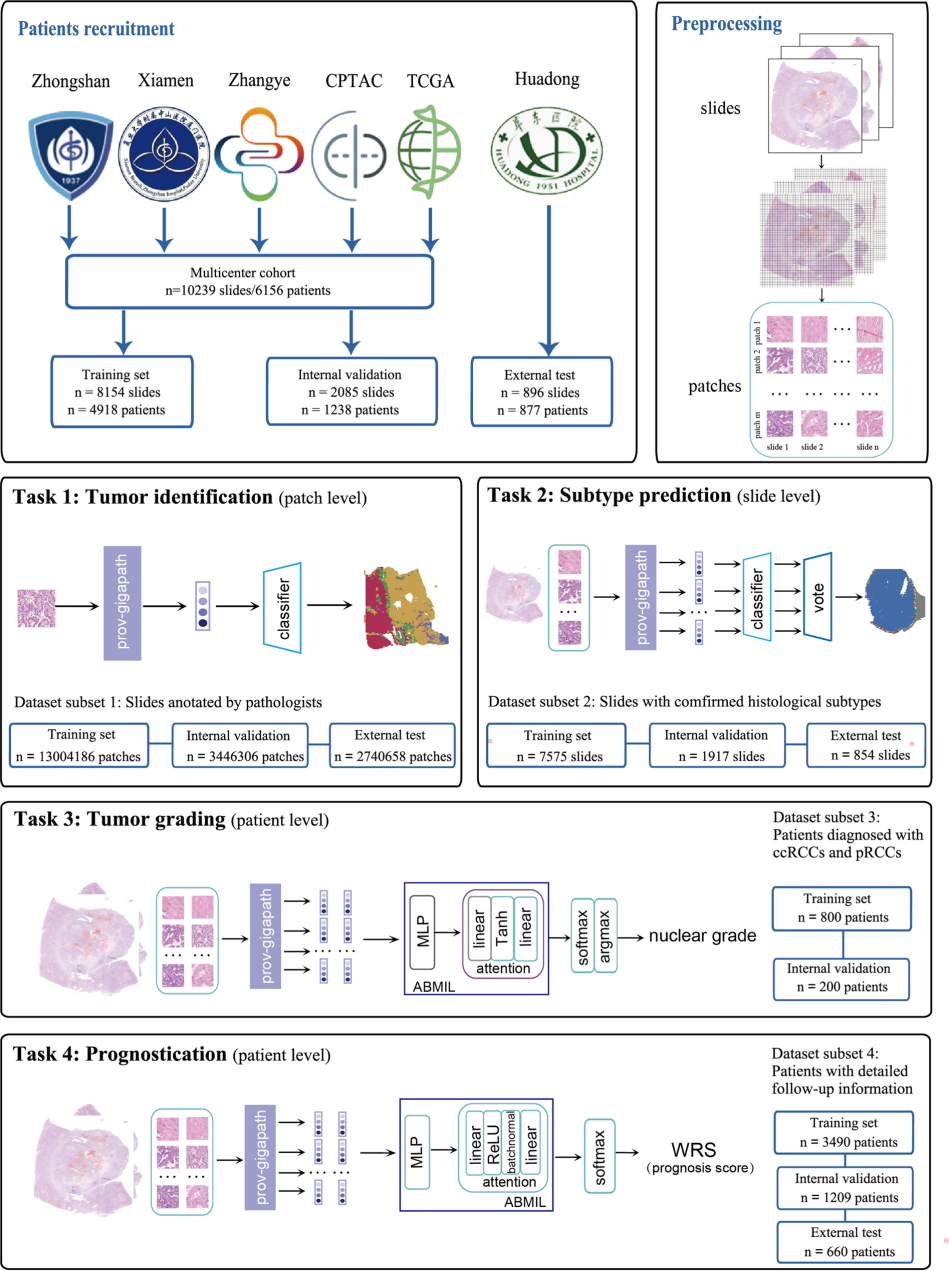
Patient recruitment and overview of the study. A total of 10,239 whole-slide images (WSIs) from 6,156 patients were collected from five medical centers for training and internal validation. The cohort was divided into a training set (*n* = 8,154 slides, 4,918 patients), an internal validation set (*n* = 2,085 slides, 1,238 patients). The Huadong cohort was used as an external test set (*n* = 896 slides, 877 patients). After preprocessing, WSIs were tiled into non-overlapping image patches. Four downstream tasks were conducted: **Task 1 (Tumor identification)**: patch-level classification of tumor regions using slides annotated by pathologists. **Task 2 (Subtype prediction)**: slide-level classification of histological subtypes for confirmed renal tumor slides. **Task 3 (Tumor grading)**: patient-level prediction of nuclear grade in clear cell and pRCC using an attention-based multiple instance learning (ABMIL) model. **Task 4 (Prognostication)**: patient-level prediction of overall prognosis using an ABMIL model to generate the WRS (prognosis score)

To develop and validate the deep learning models, we retrospectively enrolled a total of 11,135 whole-slide images (WSIs) derived from 7,033 individual patients across five cohorts: Zhongshan Hospital (8,303 WSIs/4,809 patients), Huadong Hospital (896 WSIs/877 patients), Zhangye Hospital (110 WSIs/110 patients), Xiamen Hospital (222 WSIs/141 patients), and the Cancer Imaging Archive (TCIA) cohort (1,604 WSIs/1,096 patients). Cohort allocation was standardized as follows: 80% of patients from Zhongshan Hospital, Zhangye Hospital, Xiamen Hospital, and the TCIA cohort (TCGA-KIRC, TCGA-KIRP, TCGA-KICH, and CPTAC-CCRCC were collected for the TCIA cohort) were assigned to the training cohort, with the remaining 20% of these patients forming the validation cohort, while patients from Huadong Hospital were exclusively designated as the test cohort. This cohort distribution was consistently applied across all four subsequent diagnostic tasks, including tissue component detection, pathologic subtype classification, grading of ccRCC and pRCC, and prediction of prognosis. All cohorts are detailed in Additional file 1: Table 1. Graphic abstract of cohort information and study design was demonstrated in Fig. 1.

### Automated detection of tissue components in pan-renal cell tumor slides

**Fig. 2:**
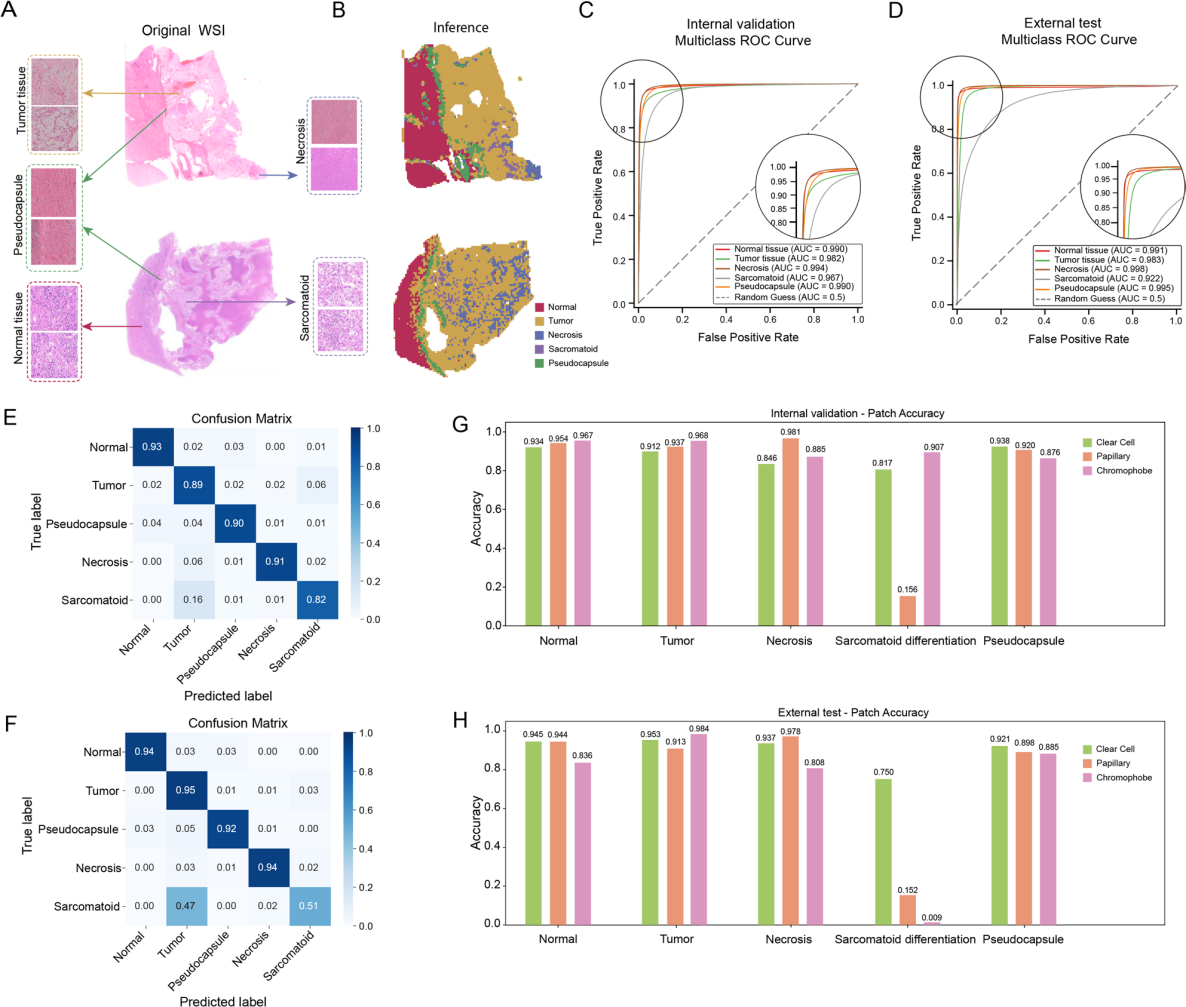
Detection of different tissue components in pan-renal cell tumor slides.** A**, Representative examples of two whole-slide images containing five distinct tissue regions. For each slide, representative patches corresponding to the five tissue components are shown. **B**, Model inference results on two representative whole-slide images. Different colors indicate distinct tissue categories predicted by the model. **C**,** D**, Receiver operating characteristic (ROC) curves of the five tissue components in the internal validation cohort (**C**) and external test cohort (**D**). **E**,** F**, Confusion matrices of the internal validation (**E**) and external test (**F**) cohorts demonstrating high consistency between predicted and true tissue labels. **G**,** H**, Prediction accuracy of five tissue components across three major renal cancer subtypes in the internal validation and external test cohorts

For the task of tissue component detection, which focused on identifying five distinct tissue components (normal tissue, tumor tissue, pseudo capsule, necrosis, and sarcomatoid differentiation), WSIs were manually annotated for these five regions by four experienced pathologists, and model performance was primarily evaluated based on patch-level predictive accuracy. To enable accurate identification of tissue components in pan-RCC slides, we first established a category-based sampling strategy by training a classification network targeting five key RCC tissue components (Fig. 2A). Typical visualization results of the classification network output are presented in Fig. 2B, where distinct tissue components are labeled with different colors for intuitive distinction. The total number of image patches used for network training is detailed in Additional file 1: Table 3. The classification network exhibited robust performance across all tissue components, achieving high area under the receiver operating characteristic curve (AUC) values in both the validation and test sets: 0.990 (validation) and 0.991 (test) for normal tissue; 0.982 (validation) and 0.983 (test) for tumor tissue; 0.994 (validation) and 0.998 (test) for necrosis; and 0.967 (validation) and 0.922 (test) for sarcomatoid differentiation; 0.990 (validation) and 0.995 (test) for pseudo capsule (Fig. 2C and D). Confusion matrices further verifying the network’s classification accuracy are provided for the validation set (Fig. 2E) and test set (Fig. 2F), respectively. For a comprehensive assessment of performance, detailed metrics including sensitivity, specificity, and F1 score in both the validation and test sets are summarized in Additional file 1: Table 4. We further evaluated the tissue discrimination capability of the classification network using the t-distributed stochastic neighbor embedding (t-SNE) algorithm, which revealed clear clustering and separation among the four tissue, except for sarcomatoid tissue, across the Zhongshan Hospital, TCGA, and Huadong Hospital cohorts (Additional file 1: Fig. S1). This finding is consistent with the observation that sarcomatoid differentiation tissue is the most challenging areas for the model to distinguish. Notably, the network maintained accurate tissue component identification across different RCC pathologic subtypes, as illustrated in Fig. 2G and H.

### Classification of nine renal cell tumor histologic subtypes

**Fig. 3:**
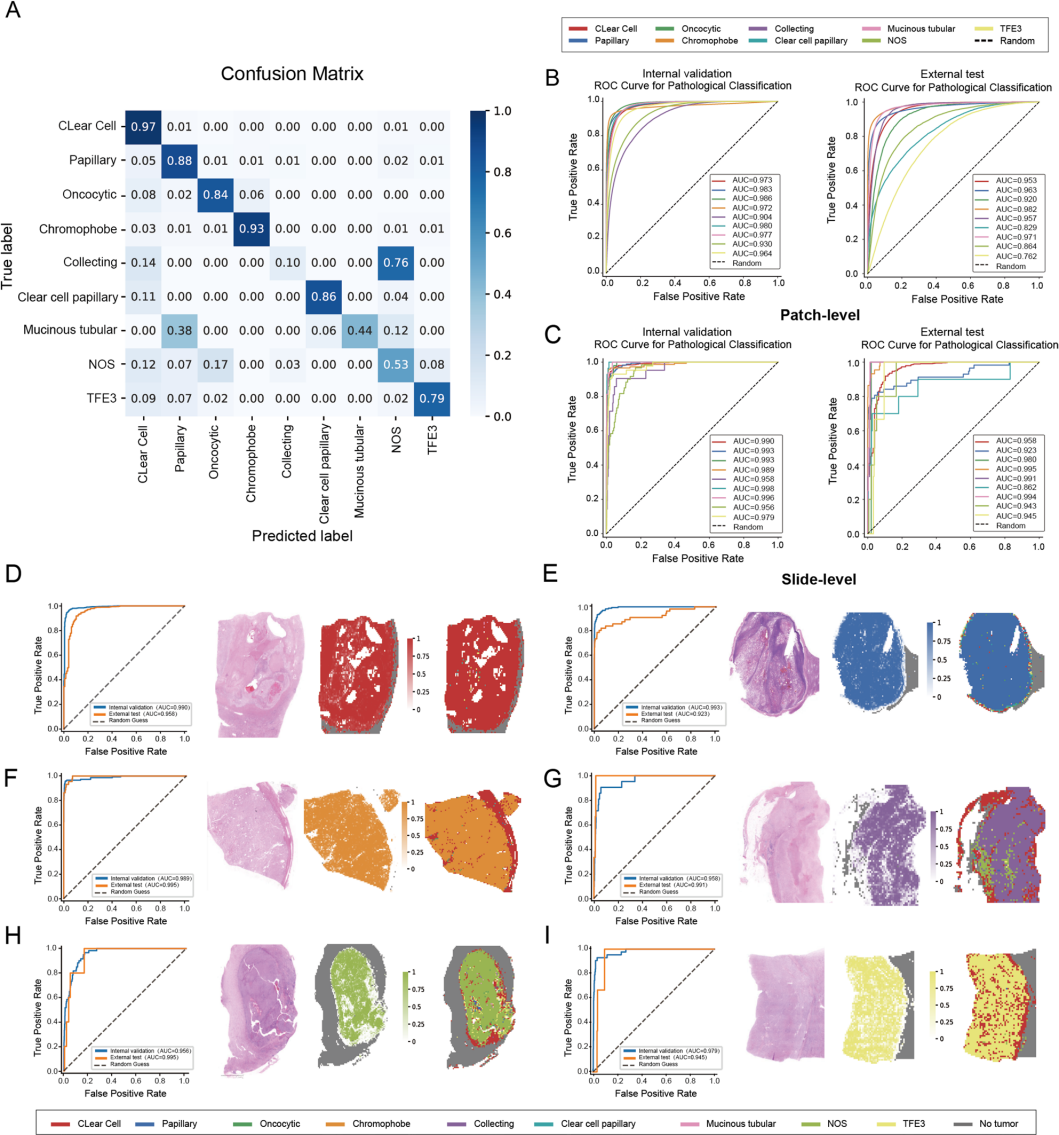
Deep learning model for classification of nine renal tumors. The model first predicts the subtype for each patch, and the final slide-level subtype is determined by the majority subtype among all predicted patches. **A**, Confusion matrix of nine RCC subtypes in the internal validation cohort. **B**, Patch-level and slide-level ROC curves for nine subtypes in both the internal validation and external test cohorts. **C-H**, Representative visualization results for six selected subtypes, including the ROC curve, corresponding original whole-slide image, probability heatmap, and spatial distribution of predicted patch subtypes

For the pathological subtype classification of renal tumor, only renal cell tumor subtypes with a sample size exceeding 10 patients were included to ensure statistical robustness. Model performance was primarily evaluated using slide-level predictive accuracy. A total of nine histological subtypes were included in the classification analysis. For ccRCC, pRCC, renal oncocytoma, chRCC, clear cell papillary renal cell tumor, mucinous tubular and spindle cell carcinoma, and collecting duct carcinoma, the reference diagnoses were established primarily on the basis of morphological features, supported by appropriate immunohistochemical (IHC) findings. FISH and next-generation sequencing (NGS) are not routinely required for the diagnosis of these entities. TFE3(transcription factor binding to IGHM enhancer 3)-rearranged RCC was initially screened using TFE3 immunohistochemistry and definitively confirmed by TFE3 break-apart FISH, with NGS performed in selected cases when necessary. RCC NOS was diagnosed by consensus among multiple experienced pathologists only after recognized RCC entities had been excluded through comprehensive morphological evaluation, an appropriate IHC panel, and NGS when indicated. The number of patches and slides corresponding to each subtype are detailed in Additional file 1: Table 5. Confusion matrices of subtype predictions in the internal validation set and external test set are presented in Fig. 3A and Additional file 1: Fig. S2A, respectively. At the patch level, the macro-AUC was 0.963 for the internal validation set and 0.911 for the external test set; at the slide level, the macro-AUC was 0.983 for the internal validation set and 0.928 for the external test set (Fig. 3B and C). Subtype-specific slide-level AUCs were as follows: 0.990 (validation) and 0.958 (test) for ccRCC; 0.993 (validation) and 0.923 (test) for pRCC; 0.993 (validation) and 0.980 (test) for renal oncocytoma; 0.989 (validation) and 0.995 (test) for chromophobe RCC; 0.958 (validation) and 0.991 (test) for collecting duct carcinoma; 0.998 (validation) and 0.862 (test) for clear cell papillary renal cell carcinoma; 0.996 (validation) and 0.994 (test) for mucinous tubular and spindle cell carcinoma; 0.956 (validation) and 0.943 (test) for RCC, not otherwise specified (NOS); and 0.979 (validation) and 0.945 (test) for TFE3-rearranged RCC (Fig. 3D-3I, Additional file 1: Fig. S2B-S2D). A sliding window approach was employed to visualize the classification results of all subgroups and the classification probability of the most prevalent subgroup on representative slides (Fig. 3D-3I, Additional file 1: Fig. S2B-S2D). Patch-level performance measures are provided in Additional file 1: Table 6. At the slide level, the classification performance for the three most common renal tumor subtypes varied across cohorts. In the internal validation cohort, ccRCC (931 patients/1,381 slides) achieved a sensitivity of 0.975, specificity of 0.937, PPV of 0.975, NPV of 0.935, F1 score of 0.975, and PR AUC of 0.996; pRCC (122 patients/172 slides) achieved corresponding values of 0.884, 0.985, 0.854, 0.988, 0.869, and 0.955; and chRCC (96 patients/146 slides) achieved values of 0.932, 0.994, 0.932, 0.994, 0.932, and 0.961, respectively. In the external test cohort, the corresponding sensitivity, specificity, PPV, NPV, F1 score, and PR AUC were 0.992, 0.709, 0.951, 0.938, 0.971, and 0.991 for ccRCC (726 patients/727 slides); 0.649, 0.996, 0.925, 0.975, 0.763, and 0.819 for pRCC (57 patients/57 slides); and 0.795, 1.000, 1.000, 0.989, 0.886, and 0.946 for chRCC (44 patients/44 slides), respectively. Complete per-class results for all nine subtypes are provided in Additional file 1: Table 7. Additionally, we evaluated a comprehensive four-way classification model by pooling all rare subtypes into a single ‘other’ category. This model successfully discriminated common subtypes while effectively flagging out-of-distribution cases that require further human oversight and molecular testing; detailed performance for both cohorts are available in Additional file 1: Fig. S3.

### Auto-grading of clear cell renal cell carcinoma and papillary renal cell carcinoma

**Fig. 4:**
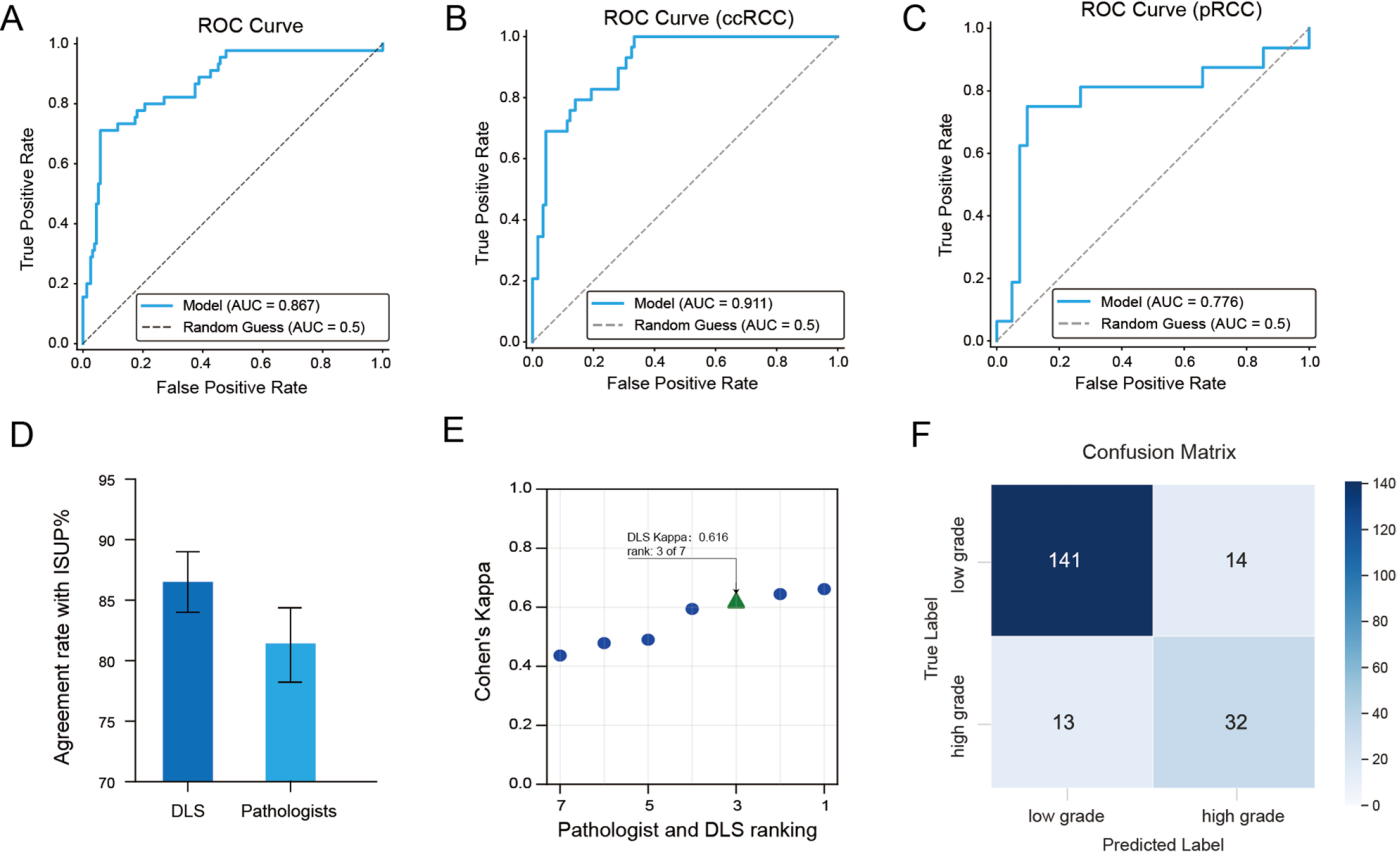
Auto-grading of ccRCC and pRCC.** A**, ROC curve of the validation cohort. **B**, ROC curve for patients with ccRCC in the validation cohort. **C**, ROC curve for patients with pRCC in the validation cohort. **D**, Agreement between the DLS and the pathological gold standard, as well as the mean agreement of six pathologists with the gold standard, 95% confidence intervals estimated by the bootstrap method (*n* = 1000 times). **E**, Comparison of Cohen’s kappa values among the model and six pathologists. **F**, Confusion matrix of the DLS in the validation cohort

The WHO/ISUP grading system for RCC has achieved relatively widespread acceptance in clinical practice; however, uncertainties persist regarding the degree of nucleolar prominence that justifies assigning a higher nuclear grade. These ambiguities contribute to persistent challenges in RCC grading: a prior study demonstrated that the interobserver kappa agreement for RCC grading between two pathologists was merely 0.45, highlighting substantial subjective variability in manual grading. To develop and validate an AI-based nuclear grading prediction model for RCC, a cohort of whole-slide images (WSIs) was strategically selected. Specifically, the training set comprised 600 cases of clear cell RCC (ccRCC) and 200 cases of papillary RCC (pRCC), while the validation set included 143 ccRCC cases and 57 pRCC cases. The number of grade 1–4 to ccRCC and pRCC are detailed in Additional file 1:Table 8. This targeted cohort size warrants an explicit explanation, as establishing a rigorous grading ground truth is highly resource-intensive. Given the high heterogeneity inherent in RCC nuclear grading, all selected WSIs underwent independent review by a panel of three subspecialized expert urologic renal pathologists. Because this meticulous consensus process requires substantial expert labor and time, it was only feasible to execute it on this curated subset. Ultimately, a unanimous consensus on nuclear grade assignments was reached among the experts, and this consensus was adopted as the gold standard for evaluating model performance. Notably, model performance was assessed using patient-level predictive accuracy to ensure clinical relevance. ROC curve analyses were conducted to evaluate the model’s discriminative ability at a clinically critical decision threshold: distinguishing low-grade RCC (grades 1–2) from high-grade RCC (grades 3–4). In the validation set, the deep learning system (DLS) achieved an AUC of 0.867 for differentiating high-grade from low-grade RCC overall (Fig. 4A). Subgroup analyses further revealed that the DLS exhibited an AUC of 0.911 for ccRCC and 0.776 for pRCC, respectively (Fig. 4B and C). Detailed metrics including accuracy, specificity, and sensitivity are presented in Additional file 1: Table 9. Crucially, the DLS demonstrated superior performance compared to general pathologists in aligning with expert consensus grades for both ccRCC and pRCC (Fig. 4D ~ 4E). The agreement rate between the DLS and subspecialized experts was 86.4% (95% confidence interval [CI], 81.5%-91.0%), which was significantly higher than the agreement rate between general pathologists and subspecialized experts 81.4% (95% CI, 78.2%-84.5%) (*P* < 0.001) (Fig. 4D). When individually compared to 6 general pathologists, the DLS outperformed 4 of them in terms of agreement with expert consensus grades (Fig. 4E). Confusion matrix in validation cohort is presented in Fig. 4F. After training and validation on a cohort of 1,000 patients, the model was applied to all cases of ccRCC and pRCC to evaluate the prognostic ability of the predicted nuclear grading. We observed a significant survival disadvantage for patients categorized as high-grade compared to their low-grade counterparts, thereby validating the clinical predictive power of our automated nuclear grading system (Additional file 1: Fig. S4-S7). To comprehensively evaluate the model’s performance on this imbalanced dataset, we report the area under the precision-recall curve (AUPRC). The model achieved an AUPRC of 0.697(Additional file 1: Fig. S8). Notably, the AUPRC substantially exceeded the baseline of random chance (equivalent to the positive-class prevalence of 0.225) by approximately 3.1-fold, demonstrating robust precision-recall performance despite the data imbalance.

### WSI-based risk score predicting oncologic outcomes for pan-renal cell tumor patients

**Fig. 5:**
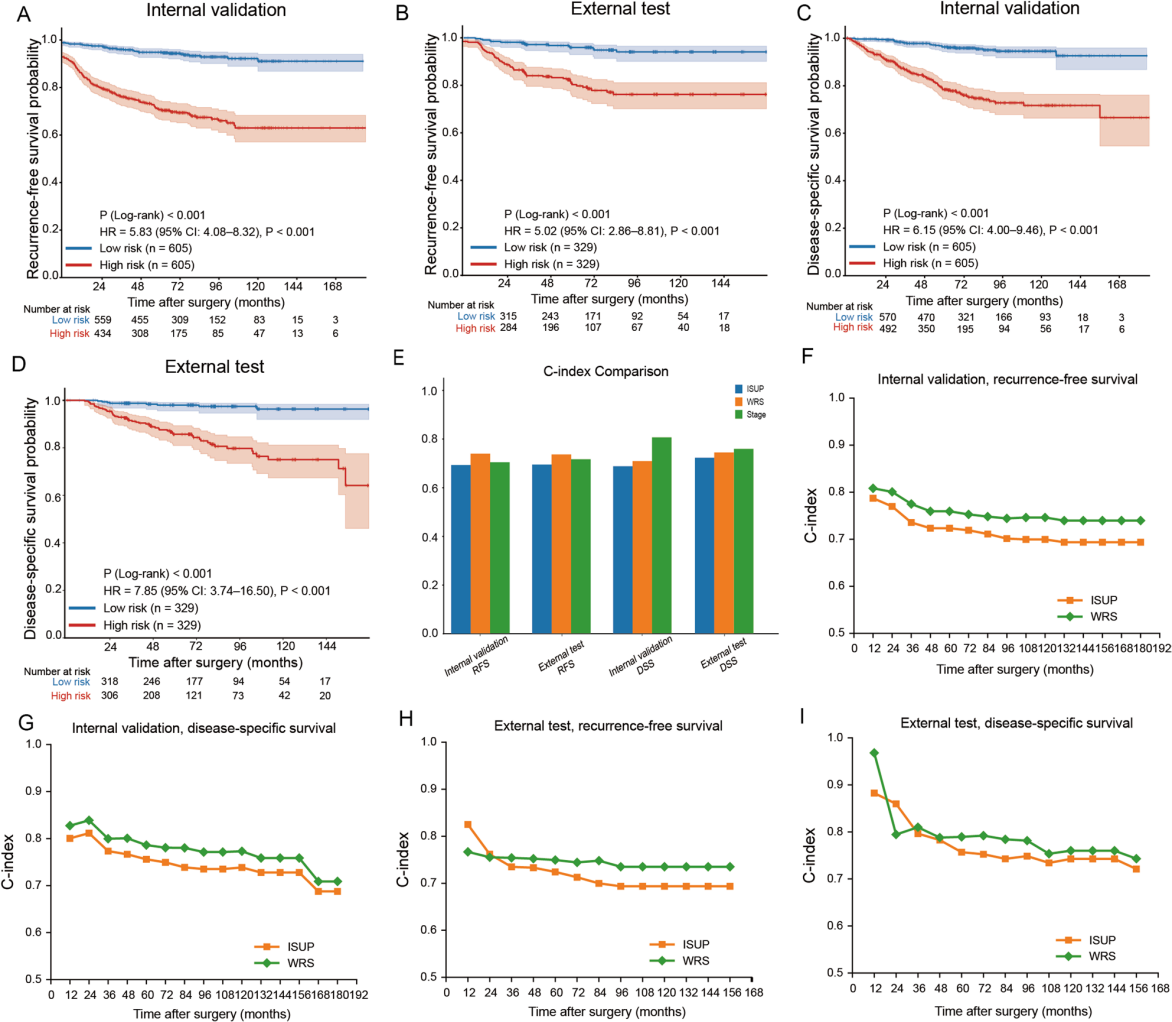
Prognostic network predicting oncologic outcomes for pan-renal tumor patients.** A**,** B** Kaplan-Meier survival curves of recurrence-free survival (RFS) based on WRS scores in the internal validation cohort (**A**) and the external test cohort (**B**). **C**,** D** Kaplan-Meier survival curves of disease-specific survival (DSS) based on WRS scores in the internal validation cohort (**C**) and the external test cohort (**D**). **E** Comparison of concordance indices (C-index) among WRS score, ISUP grade, and TNM stage across internal and external cohorts for both RFS and DSS outcomes. **F**,** G** Time-dependent C-index comparison between WRS score and ISUP grade for RFS in the internal (**F**) and external (**G**) cohorts. **H**,** I** Time-dependent C-index comparison between WRS score and ISUP grade for DSS in the internal (**H**) and external (**I**) cohorts

For prognostic assessment, our study exclusively incorporated patients with accessible clinical outcome data, encompassing recurrence-free survival (RFS), disease-specific survival (DSS), or overall survival (OS). In the training dataset, patients who experienced recurrence during the follow-up period were classified as having a poor prognosis. Conversely, patients who remained recurrence-free throughout the entire follow-up period and had a follow-up duration exceeding 5 years were categorized as having a good prognosis. A total of 3490 patients from the training set exhibited distinct outcomes (either good or poor prognosis), providing a clear ground truth for our analysis. The number of patches, slides, and patients corresponding to each subtype are detailed in Additional file 1: Table 10. We developed a deep learning-based risk score, termed the WSI-based Risk Score (WRS), by employing deep learning models to predict patients with a poor prognosis. Tumors classified as high-risk based on the WRS (with the median value as the cutoff) demonstrated significantly worse RFS, DSS, and OS in both the internal validation and external test cohorts (Fig. 5A and D, Additional file 1: Fig. S9A-S9B). Furthermore, we compared the concordance index (C-index) of the WRS with that of different histopathological data provided by pathologists, including WHO/ISUP grade and TNM stage. Although the WRS exhibited somewhat limited performance compared to the TNM stage, which incorporates information not inferable from tissue images, such as tumor size and fat invasion, it outperformed the WHO/ISUP grade across all three survival events (Fig. 5E, Additional file 1: Fig. S9C-S9D). Specifically, the C-index for RFS was 0.740 for WRS versus 0.692 for grade in internal validation cohort, 0.735 for WRS versus 0.694 for grade in external test cohort; for DSS, it was 0.709 for WRS versus 0.689 for grade in internal validation cohort, 0.743 for WRS versus 0.721 for grade in external test cohort. Notably, at each time point from 1 to 10 years, the WRS consistently exhibited superior prognostic ability, as evidenced by higher C-index values for RFS, DSS, and OS (Fig. 5F and I, Additional file 1: Fig. S9E-S9F). Recognizing that the WHO/ISUP grading system is clinically validated and standardized specifically for clear cell (ccRCC) and papillary (pRCC) subtypes, we conducted a restricted subgroup analysis to prevent conflating the prognostic value of the WRS. When restricted exclusively to the ccRCC and pRCC cohorts, the continuous WRS demonstrated a higher concordance index (C-index) than the routine ISUP grade across all evaluated endpoints: recurrence-free survival (0.718 vs. 0.661) disease-specific survival (0.709 vs. 0.690), and overall survival (0.839 vs. 0.829). Furthermore, time-dependent C-index analysis confirmed that the discriminative superiority of the WRS over ISUP grading was maintained consistently throughout the follow-up period (Additional file 1: Fig. S10). This underscores the potential of the WRS as a robust and clinically relevant tool for predicting oncologic outcomes in pan-renal cell tumor patients. WRS remained an independent adverse risk factor in the validation and test cohorts for both RFS and DSS after adjusting for stage, grade, necrosis and sarcomatoid differentiation Additional file 1: Tables 11, 12, 13 and 14. Furthermore, an alternative analysis utilizing WRS quartiles demonstrated a highly significant, monotonic risk gradient across all endpoints, confirming our findings are not an artifact of the median split (Additional file 1: Fig. S11). Furthermore, the WRS was evaluated as a continuous variable without any thresholding via Cox proportional-hazards regression (Additional file 1: Table 15).

## Discussion

The development of computer vision and digital pathology has led to increased interest in application of deep learning based artificial intelligence in tumor pathology [30, 31]. Recent studies showed that deep learning algorithms could conduct all kinds of pathologic work including diagnosis, subtyping, grading, and prognostic prediction, with accuracies equivalent to or better than experienced pathologists [8]. They not only help reduce workload of pathologists and enable them to spend more time on complicated decision-making tasks, but also show great potential to improve accuracy, objectivity, consistency, and reproducibility of diagnosis. In this study, we developed for the first time a “full-stack” smart diagnosis system for renal cell tumors, which includes identification, classification, grading and survival prediction.

For renal cell tumors, several previous studies explored the ability of deep learning algorithms to distinguish RCC from normal tissue. Tabibu et al. developed a diagnostic model which achieved region-wise accuracy of 93.62% for ccRCC and 80.57% for chRCC (slide-wise AUC of 0.99 for ccRCC and 0.95 for chRCC) [32]. The slide-wise AUC of a Lasso diagnostic model proposed by Chen et al. was 0.96 for ccRCC, 0.945 for pRCC and 0.876 for chRCC [17]. Another previous study using ResNet-50 showed the highest region wise AUCs of 0.953 for ccRCC, 0.991 for pRCC and 0.953 for chRCC (slide-wise AUC of 0.99 for KIRC and 0.95 for KICH) [16]. The region-wise AUC in independent validation set was 0.919 (slide-wise AUC of 0.970). All three studies trained their models based on small number of cases from TCGA-KIRC, TCGA-KIRP, and TCGA-KICH cohorts only. The identification models can be applied to three subtypes of renal cell tumors, which severely limited their applications. On the contrary, we included WSIs of 7,033 patients with pan-renal cell tumors from four medical centers and two public cohorts (TCGA cohorts and CPTAC cohorts). The deep learning model differentiated tumoral area, necrosis, sarcomatoid differentiation, normal tissue and pseudo capsule with AUCs of 0.982, 0.994, 0.967, 0.990, 0.990 respectively in validation cohort. In external test cohort, the AUCs for the five categories were 0.983, 0.998, 0.922, 0.991, 0.995 respectively. Necrosis, sarcomatoid differentiation and pseudo capsule had important prognostic values, which were neglected in previous studies, and our algorithms were the first to accurately identify these regions.

For tumor classification, we included 9 subtypes of renal cell tumors according to the fifth edition of WHO classifications of kidney cancer with 10,346 WSIs from 6,979 patients. The AUC is 0.973 for ccRCC, 0.983 for pRCC, 0.972 for chRCC, 0.986 for oncocytoma, 0.980 for clear cell papillary renal cell tumor, 0.904 for collecting duct carcinoma, 0.977 for mucinous tubular and spindle cell carcinoma, 0.930 for RCC, NOS, 0.964 for TFE3-rearranged RCC in validation cohort. In earlier studies, Marostica et al. constructed deep convolutional neural network models to classify ccRCC, pRCC, and chRCC, with a multi-class AUC of 0.926 with VGG-16, 0.897 with Inception-v3, and 0.953 with ResNet-50 [16]. When limited to these three subtypes, our classification model reached a multi-class AUC of 0.976. Two other models proposed by Chen et al. and Tabibu et al. had lower AUC differentiating ccRCC from pRCC/chRCC with AUCs of 0.814 and 0.93, respectively [17, 32].

Grading of renal cell tumors has been a time-consuming and challenging task due to difficulties in consistency with the four-tier classification described in the 2012 ISUP Vancouver conference and recommended by WHO [19]. The WHO/ISUP grading system showed better reproducibility and consistency than the Fuhrman grading system, but high inter-observer disagreement still remained [33]. WHO/ISUP grading is based upon the highest grades present within a high-power field of the tumor, which is subject to individual pathologist’s preferences and evaluation. Some high-grade tumors, if very focal, are easily neglected in the busy daily work [34]. Our diagnostic model reached an AUC of 0.867, and showed comparable accuracy among the most experienced pathologists. One major purpose of the grading system is to evaluate aggressiveness of renal cell tumors, and the model-predicted grades showed prognostic associations comparable to those of pathologist-assigned grades in the retrospective cohorts, supporting further investigation of AI-assisted grading as a potential decision-support approach.

However, the validity of WHO/ISUP grading system has been confirmed in ccRCC and pRCC only [35–37]. It merely evaluates tumor cell nucleoli and does not take into account other prognostic pathological features such as necrosis [38]. What’s more, more than 25 subtypes of renal cell tumors and four nuclear grades for ccRCC and pRCC exist in WHO classifications, currently there are no pathologic prognostic models that combine information of subtypes and grades together. In the era of digital pathology, which contains far more information beyond interpretation of human pathologists, there is an urgent need and opportunity to develop a comprehensive pan-renal cell tumor prognostic model based on WSIs. In this study, we developed and validated the first pan-renal cell tumor model based on WSI using deep learning. In this way, we could take into consideration all prognostic pathologic information such as tumor subtype, grading, and pathologic features. We extracted information of prognostic morphology underlying tumor subtypes and tumor nucleus grades. It showed prognostic value with C-indices for RFS of 0.740 and 0.735 in pan-renal cell tumor internal validation cohort and external test cohort, respectively, significantly higher than WHO/ISUP grading system (*p* < 0.001).

Some limitations remain in our study. By far, we have included the most cases and subtypes of renal cell tumors, but there were still some rare subtypes missing due to limited cases for training and validating the model. The full spectrum of histological heterogeneity could not be fully captured. Another limitation was that we did not perform prospective validations. Before translation into clinical application, the deep learning algorithms need to be further validated in prospective clinical trials. Moreover, there are many new entities in the fifth edition of WHO classification of urogenital tumors, especially molecularly defined renal tumor entities. Even though three experienced urological pathologists re-evaluated the slides and pathological reports according to the fifth edition, some rare subtypes are likely to be misclassified since most cases were originally evaluated according to the fourth edition of WHO classification. Necessary genetic or immunohistochemical examination may be absent. However, from another perspective, this more accurately reflects real-world clinical scenarios, where not all patients are willing to afford the expenses of extra tests, and not all pathologists are aware of the corresponding genetic tests.

## Conclusions

In conclusion, we presented an innovative and comprehensive deep learning framework for identification, classification, grading, and prognostication in renal cell tumors based on histology slides. Our model for identification could differentiate tumoral area, necrosis, sarcomatoid differentiation, and benign area in renal cell tumors with macro-AUCs of 0.985 and 0.978 in the internal validation and external test cohorts, respectively. The classification model achieved macro-averaged AUCs of 0.984 and 0.955 in the internal validation and external test cohorts, respectively, and showed strong discrimination for several common renal tumor subtypes. However, performance estimates for rare subtypes were limited by small external sample sizes and should therefore be interpreted as exploratory. The grading deep learning algorithm showed comparable accuracy with most experienced pathologists. In the end, we developed the first pan-renal cancer pathological scoring system for survival prediction, which outperformed the WHO/ISUP grading system. The proposed framework should be considered a potential decision-support approach rather than a clinically validated autonomous diagnostic system. Prospective studies incorporating real-world deployment and pathologist-AI interaction are required to determine whether it improves diagnostic accuracy, interobserver agreement, reporting efficiency, or pathologist workload. Within the retrospective cohorts, the AI-derived risk score provided additional prognostic information beyond conventional histological assessment.

## Supplementary Information

Below is the link to the electronic supplementary material.

Supplementary Material 1: Additional file 1: Figures S1 - S11, Tables 1 - 15

## Data Availability

The source code and pre-trained models are publicly available at https://github.com/Green8023/full-stackRCC. Due to ethical mandates and patient confidentiality protocols, the datasets are not publicly accessible. Specifically, whole-slide images (WSIs) encompass highly sensitive, gigapixel-level morphologic data. Publicly exposing such individualized, high-resolution information carries unforeseen risks of patient re-identification and contravenes institutional privacy guidelines. The data are available from the corresponding author on reasonable request shuowang10@fudan.edu.cn. Upon receiving a request, we will take one to two weeks to evaluate the intended use, the requester’s qualifications, and ethical compliance. After evaluation, we will contact the requester within one week to discuss the terms and arrangements for data access.

## Abbreviations

ABMIL: attention-based multiple instance learning
AI: artificial intelligence
AJCC: American Joint Committee on Cancer
AUC: area under the receiver operating characteristic curve
AUPRC: area under the precision–recall curve
ccRCC: clear cell renal cell carcinoma
CFFF: Computing for the Future at Fudan
chRCC: chromophobe renal cell carcinoma
CI: confidence interval
C-index: concordance index
CPTAC: Clinical Proteomic Tumor Analysis Consortium
CPTAC-CCRCC: Clinical Proteomic Tumor Analysis Consortium clear cell renal cell carcinoma cohort
DINOv2: self-distillation with no labels, version 2
DLS: deep learning system
DSS: disease-specific survival
F1: score: harmonic mean of precision and recall
H&E: hematoxylin and eosin
ISUP: International Society of Urological Pathology
KICH: Kidney Chromophobe
KIRC: Kidney Renal Clear Cell Carcinoma
KIRP: Kidney Renal Papillary Cell Carcinoma
MLP: multilayer perceptron
NOS: not otherwise specified
OS: overall survival
pRCC: papillary renal cell carcinoma
RCC: renal cell carcinoma
ReLU: rectified linear unit
RFS: recurrence-free survival
ROC: receiver operating characteristic
SGD: stochastic gradient descent
SPSS: Statistical Package for the Social Sciences
TCGA: The Cancer Genome Atlas
TCIA: The Cancer Imaging Archive
IHC: immunohistochemical
NGS: next-generation sequencing
TFE3: transcription factor binding to IGHM enhancer 3
TNM: tumor–node–metastasis
t-SNE: t-distributed stochastic neighbor embedding
ViT: Vision Transformer
WHO/ISUP: World Health Organization/International Society of Urological Pathology
WRS: whole-slide image-based risk score
WSI: whole-slide image
