# Supplementary material for: Artificial intelligence empowers full-stack histopathological diagnosis and prognosis of renal cell tumor: a multi-center study with external validation
Source: BMC Med. 2026 Aug 4;24:408. doi: 10.1186/s12916-026-05110-5 (PMC13435775; doi:10.1186/s12916-026-05110-5)
Supplement: Supplementary file 1 [file 12916_2026_5110_MOESM1_ESM.docx]

**Supplementary Materials**

**Artificial intelligence empowers full-stack smart diagnosis of renal cell tumors**

**Contents**

[Fig.S1: t-SNE visualization of features across data centers.	4](#_Toc24033)

[Fig.S2: Supplementary map of pathological type classification.	5](#_Toc24464)

[Fig.S3: Kaplan–Meier curves of recurrence-free survival (RFS) in the internal validation cohort.	6](#_Toc3612)

[Fig.S4: Kaplan–Meier curves of Disease-specific survival (DSS) in the internal validation cohort.	7](#_Toc27742)

[Fig.S5: Kaplan–Meier curves of recurrence-free survival (RFS) in the external test cohort.	8](#_Toc17885)

[Fig.S6: Kaplan–Meier curves of Disease-specific survival (DSS) in the external test cohort.	9](#_Toc1728)

[Fig.S7: Kaplan–Meier survival analysis and time-dependent C-index comparison of the WRS score and ISUP grading.	11](#_Toc7521)

[Fig.S8. Prognostic evaluation of the AI-derived risk score (WRS), ISUP grading, and TNM staging in the combined cohort (ccRCC and pRCC).	12](#_Toc18766)

[Table 1: Clinicopathological information of patients and number of whole slide images included in this study	13](#_Toc17162)

[Table 2: Number of image patches and slides analyzed for tumor identification	15](#_Toc13945)

[Table 3: Sensitivity, specificity and F1 score for tissue detection task	17](#_Toc28340)

[Table 4: Number of image patches and slides analyzed for subtype prediction	18](#_Toc12569)

[Table 5: Patch-level sensitivity, specificity and F1 score for classification of nine renal cell carcinoma	19](#_Toc156)

[Table 6: Slide-level sensitivity, specificity and F1 score for classification of nine renal cell carcinoma	20](#_Toc1586)

[Table 7: Number of patients and slides analyzed for WHO/ISUP grades prediction	21](#_Toc12418)

[Table 8: Sensitivity, specificity and F1 score for nuclear grading task	22](#_Toc31243)

[Table 9: Clinicopathological information of patients with follow-up information across different cohorts	23](#_Toc5878)

[Table 10: Univariate analysis and Multivariate analysis of recurrence-free survival in internal validation cohort	25](#_Toc6862)

[Table 11: Univariate analysis and Multivariate analysis of disease-specific survival in internal validation cohort	26](#_Toc27119)

[Table 12: Univariate analysis and Multivariate analysis of recurrence-free survival in external test cohort	27](#_Toc27971)

[Table 13: Univariate analysis and Multivariate analysis of disease-specific survival in external test cohort	28](#_Toc20242)

[1. Methods	29](#_Toc27760)

[1.1 Study cohorts and data preprocessing	29](#_Toc31074)

[1.1.1 Study population	29](#_Toc23326)

[1.1.2 Patient cohort	29](#_Toc13604)

[1.1.3 WSIs acquisition and preprocessing	30](#_Toc7824)

[1.2 Main model architecture	30](#_Toc23619)

[1.2.1 Feature extract	30](#_Toc21491)

[1.2.2 Aggregation	31](#_Toc15994)

[1.3 Downstream task model	31](#_Toc7759)

[1.3.1 Tissue detection	31](#_Toc9219)

[1.3.2 Classification of nine renal cell carcinomas	32](#_Toc22785)

[1.3.3 Nuclear grading	32](#_Toc1703)

[1.3.4 Prognosis prediction	33](#_Toc31495)

[1.4 Evaluation of downstream tasks	34](#_Toc15453)

[1.4.1 Evaluation metrics	34](#_Toc6565)

[1.4.2 Statistical analysis	34](#_Toc26592)

[1.4.3 Computing hardware and software	34](#_Toc11559)

[References:	35](#_Toc26332)

# Fig.S1: t-SNE visualization of features across data centers. After feature extraction, t-SNE analysis was performed on the three primary datasets. For each dataset, 5,000 feature vectors were randomly sampled from each of the five tissue types, yielding a total of 25,000 patch features per dataset. Distinct colors were used to visualize and differentiate the five tissue categories. A, Features from the Zhongshan Hospital of Fudan University. B, Features from the Huadong Hospital Affiliated to Fudan University. C, Features from the public TCGA dataset.

# Fig.S2: Supplementary map of pathological type classification. A, Confusion matrix of external test cohort. B, ROC curves, probability heatmaps, and patch-level subtype distribution visualizations for chromophobe RCC. C, ROC curves, probability heatmaps, and patch-level subtype distribution visualizations for Clear cell papillary renal cell carcinoma. D, ROC curves, probability heatmaps, and patch-level subtype distribution visualizations for mucinous tubular and spindle cell carcinoma.

# Fig.S3. Supplementary analysis of renal tumor classification with a pooled "rare/other" lump class. To evaluate model applicability across the full spectrum of renal tumors, all rare subtypes were aggregated into a single pooled "Other" class, yielding a four-way classification comprising the three most common subtypes (ccRCC, pRCC, and chRCC) and the combined rare class.

# Fig.S4: Kaplan–Meier curves of recurrence-free survival (RFS) in the internal validation cohort. After training and validation on a cohort of 1,000 patients, the model was applied to all cases of ccRCC and pRCC. The resulting Kaplan–Meier survival curves closely mirrored those generated using ISUP binary grading (low vs high grade), indicating that the model’s classification performance approximated the diagnostic level of ISUP grading. A, RFS curves based on model-predicted grade for ccRCC and pRCC, ccRCC only, and pRCC only. B, RFS curves based on ISUP grading for all patients, ccRCC only, and pRCC only.

# Fig.S5: Kaplan–Meier curves of Disease-specific survival (DSS) in the internal validation cohort. After training and validation on a cohort of 1,000 patients, the model was applied to all cases of ccRCC and pRCC. The resulting Kaplan–Meier survival curves closely mirrored those generated using ISUP binary grading (low vs. high grade), indicating that the model’s classification performance approximated the diagnostic level of ISUP grading. A, DSS curves based on model-predicted grade for ccRCC and pRCC, ccRCC only, and pRCC only. B, DSS curves based on ISUP grading for all patients, ccRCC only, and pRCC only.

# Fig.S6: Kaplan–Meier curves of recurrence-free survival (RFS) in the external test cohort. After training and validation on a cohort of 1,000 patients, the model was applied to all cases of ccRCC and pRCC. The resulting Kaplan–Meier survival curves closely mirrored those generated using ISUP binary grading (low vs high grade), indicating that the model’s classification performance approximated the diagnostic level of ISUP grading. A, RFS curves based on model-predicted grade for ccRCC and pRCC, ccRCC only, and pRCC only. B, RFS curves based on ISUP grading for all patients, ccRCC only, and pRCC only.

# Fig.S7: Kaplan–Meier curves of Disease-specific survival (DSS) in the external test cohort. After training and validation on a cohort of 1,000 patients, the model was applied to all cases of ccRCC and pRCC. The resulting Kaplan–Meier survival curves closely mirrored those generated using ISUP binary grading (low vs high grade), indicating that the model’s classification performance approximated the diagnostic level of ISUP grading. A, DSS curves based on model-predicted grade for ccRCC and pRCC, ccRCC only, and pRCC only. B, DSS curves based on ISUP grading for all patients, ccRCC only, and pRCC only.

# Fig.S8. Precision–recall analysis of slide-level nuclear grade prediction under class imbalance. Precision–recall (PR) curve for the binary high- versus low-grade prediction task in the slide-level test cohort (N = 200), in which the classes are imbalanced (high-grade has 45 and low-grade has 155).

# Fig.S9: Kaplan–Meier survival analysis and time-dependent C-index comparison of the WRS score and ISUP grading. A, Kaplan–Meier curves of overall survival (OS) in the internal validation cohort, stratified by the model-derived risk score (WRS). B, Kapla–Meier curves of OS in the external test cohort, stratified by the WRS. C, Time-dependent C-index curves comparing the prognostic performance of the WRS and ISUP grading in the internal validation. D, Time-dependent C-index curves comparing the prognostic performance of the WRS and ISUP grading in the external test cohorts.

# Fig.S10. Prognostic evaluation of the AI-derived risk score (WRS), ISUP grading, and TNM staging in the combined cohort (ccRCC and pRCC). **A, D, G,** Kaplan–Meier curve of recurrence-free survival (RFS), disease-specific survival(DSS) and overall survival (OS) stratified by the WRS. **B, E, H,** C-index comparison of ISUP grading, WRS, and TNM staging for RFS, DSS, OS prediction. **C, F, I,** Time-dependent C-index curves comparing the prognostic performance of the WRS and ISUP grading for RFS, DSS, OS.

**Fig.S11. Sensitivity analysis of the AI-derived risk score (WRS) by quartile-based stratification for survival prediction.** Patients were stratified into quartiles (Q1–Q4) according to their WRS, and survival was compared across quartiles. **A, B, C,** Kaplan–Meier curves in the internal validation cohort for recurrence-free survival, disease-specific survival, and overall survival stratified by WRS quartile. **D, E, F,** Kaplan–Meier curves in the external test cohort for RFS, DSS, and OS stratified by WRS quartile. Q1 and Q4 denote the lowest- and highest-risk quartiles, respectively; the consistent, monotonic separation across quartiles confirms that the prognostic value of the WRS is robust to the choice of threshold. P values were calculated by the log-rank test.

# Table 1: Clinicopathological information of patients and number of whole slide images included in this study

| **Variables** | **Multicenter**  **Training set**  **N=4918patients**  **/8154slides** | **Multicenter**  **test set**  **N=1238patients**  **/2085slides** | **External**  **test set**  **N=877patients**  **/896slides** |
| --- | --- | --- | --- |
| **Median age** (IQR) | 58 (66-49) | 58 (66-49) | 61 (69-53) |
| **Male sex** (%) | 3302 (67.24%) | 847 (68.34%) | 601 (68.42%) |
| **Surgical approach** (%) |  |  |  |
| Radical | 2998 (61.18%) | 734 (59.35%) | 586 (66.70%) |
| Partial | 1739 (35.46%) | 447 (36.19%) | 290 (33.07%) |
| **Pathologic tumor size** (%) |  |  |  |
| Median (IQR) | 4 (5.50-2.50) | 3.5 (5.30-2.50) | 3.5 (5.00-2.50) |
| ≤4 | 2783 (56.59%) | 731 (59.03%) | 595 (67.81%) |
| >4 to≤7 | 1339 (27.23%) | 292 (23.56%) | 203 (23.17%) |
| >7 to≤10 | 429 (8.74%) | 102 (8.26%) | 55 (6.28%) |
| >10 | 202 (4.10%) | 58 (4.70%) | 24 (2.74%) |
| **TNM stage** (%) |  |  |  |
| I | 3644 (74.34%) | 930 (75.3%) | 713 (81.16%) |
| II | 419 (8.55%) | 96 (7.69%) | 40 (4.57%) |
| III | 563 (11.47%) | 134 (10.85%) | 117 (13.36%) |
| IV | 211(4.31%) | 58(4.70%) | 7 (0.80%) |
| **WHO/ISUP Grade** (%) |  |  |  |
| 1 | 346 (7.0%) | 89 (7.21%) | 183 (20.89%) |
| 2 | 3315 (67.48%) | 834 (67.37%) | 478 (54.45%) |
| 3 | 902 (18.33%) | 221 (17.73%) | 166 (18.95%) |
| 4 | 277 (5.65%) | 73 (5.91%) | 45 (5.14%) |
| **Necrosis** (%) | 691 (14.06%) | 161 (13.04%) | 72 (8.22%) |
| **Sarcomatoid differentiation** (%) | 191 (3.90%) | 49 (3.97%) | 27 (3.08%) |
| **Medical centers** (patients/slides) |  |  |  |
| Zhongshan Hospital | 3828/6595 | 981/1708 | NA |
| Xiamen clinical cancer center | 117/186 | 24/36 | NA |
| Zhangye People’s hospital | 91/91 | 19/19 | NA |
| CPTAC-CCRCC cohort | 156/526 | 50/149 | NA |
| Huadong Hospital | NA | NA | 877/896 |
| TCGA cohorts (KIRC. KIRP, KICH) | 726/756 | 164/173 | NA |
| **Pathologic subtypes** (patients/slides) |  |  |  |
| **Clear cell renal cell tumors** |  |  |  |
| Clear cell RCC | 3723/5550 | 931/1381 | 726/727 |
| Multilocular cystic renal neoplasm of low malignant potential | 58/116 | 15/32 | 13/13 |
| **Papillary renal tumors** |  |  |  |
| Papillary RCC | 487/670 | 122/172 | 57/57 |
| **Oncocytic and chromophobe renal tumors** |  |  |  |
| Oncocytoma | 64/229 | 16/50 | 1/1 |
| Chromophobe RCC | 382/556 | 96/146 | 44/44 |
| Other oncocytic tumors of the kidney | 11/25 | 3/10 | 0/0 |
| **Collecting duct tumors** |  |  |  |
| Collecting duct carcinoma | 12/47 | 3/21 | 3/3 |
| **Other renal tumors** |  |  |  |
| Clear cell papillary renal cell tumor | 43/105 | 11/28 | 6/10 |
| Mucinous tubular and spindle cell carcinoma | 16/60 | 5/16 | 3/4 |
| Tubulocystic renal cell carcinoma | 2/2 | 1/2 | 1/3 |
| Acquired cystic disease-associated RCC | 1/2 | 0/0 | 0/0 |
| Eosinophilic solid and cystic RCC | 2/2 | 1/2 | 3/4 |
| Renal cell carcinoma, NOS | 54/192 | 14/60 | 4/5 |
| **Molecularly defined renal carcinomas** |  |  |  |
| TFE3-rearranged RCC | 46/166 | 12/43 | 3/3 |
| TFEB-altered RCC | 4/11 | 1/6 | 2/7 |
| Fumarate hydratase-deficient RCC | 1/1 | 1/5 | 2/3 |
| HLRCC syndrome-associated RCC | 1/1 | 1/1 | 1/1 |
| Medullary carcinoma, NOS | 0/0 | 0/0 | 0/0 |
| Indeterminate pathologic subtypes | 11/22 | 5/11 | 1/1 |
| **Normal slides**^1^ | 302/397 | 77/99 | 10/10 |
| 1, Patients were included in the normal slides if they had any normal slides. | | | |

# Table 2: Technical specifications of the deep-learning models

| **Task** | **Patch size** | **MPP** | **Encoder** | **Optimizer** | **Learning rate** | **Batch size** | **Epochs** | **Loss** | **Threshold strategy** | **Hardware** |
| --- | --- | --- | --- | --- | --- | --- | --- | --- | --- | --- |
| Tissue-component detection | 224 × 224 | 1 μm/pixel | Frozen Prov-GigaPath patch encoder | SGD | 1 × 10⁻⁴ | 512 | 10 | Cross-entropy loss | Youden’s J statistic | NVIDIA A100 GPU |
| Nine-subtype classification | 224 × 224 | 1 μm/pixel | Frozen Prov-GigaPath patch encoder | SGD | 1 × 10⁻³ | 512 | 10 | Cross-entropy loss | Youden’s J statistic | NVIDIA A100 GPU |
| Nuclear grading | 224 × 224 | 1 μm/pixel | Prov-GigaPath patch encoder | AdamW | 1 × 10⁻⁴ | 1 bag | 30 | Class-weighted multi-class binary cross-entropy | 0.5 | NVIDIA A100 GPU |
| Prognosis prediction | 224 × 224 | 1 μm/pixel | Frozen Prov-GigaPath patch encoder | AdamW | 1 × 10⁻⁴ | 1 bag | 20 | Cross-entropy loss | 0.5 | NVIDIA A100 GPU |
| 1. All models were implemented using Python 3.10.14, PyTorch 2.3.0, and CUDA 12.1. The Prov-GigaPath encoder remained frozen, the weights are provided by the official of Prov-Gigapath. 2. Weighted cross-entropy (class weights: 1:1:5:5:10) with class-dependent resampling in tissue-component detection task. | | | | | | | | | | |

# Table 3: Number of image patches and slides analyzed for tumor identification

| **Variables** | **Multicenter**  **Training set**  **13004186patches**  **/5319slides** | **Multicenter**  **test set**  **3446306patches**  **/1379slides** | **External**  **test set**  **2740658patches**  **/896slides** |
| --- | --- | --- | --- |
| **Tumor regions** (patches/slides) |  |  |  |
| Tumor area^1^ | 8688997/4820 | 2260358/1254 | 1799226/866 |
| Necrosis | 128951/457 | 51115/129 | 18204/43 |
| Sarcomatoid differentiation | 61486/156 | 21383/42 | 41900/24 |
| Pseudocapsule | 133253/2212 | 34050/580 | 41751/596 |
| Normal tissue | 3991499/3501 | 1079400/932 | 839577/688 |
| **Pathologic subtypes** (patches/slides) |  |  |  |
| **Clear cell renal cell tumors** |  |  |  |
| Clear cell RCC | 6688700/2720 | 1603680/675 | 2190733/727 |
| Multilocular cystic renal neoplasm of low malignant potential | 108064/116 | 39229/32 | 18117/13 |
| **Papillary renal tumors** |  |  |  |
| Papillary RCC | 1450534/669 | 409376/174 | 179453/57 |
| **Oncocytic and chromophobe renal tumors** |  |  |  |
| Oncocytoma | 696791/229 | 178212/50 | 2967/1 |
| Chromophobe RCC | 1618969/561 | 442771/147 | 178227/44 |
| Other oncocytic tumors of the kidney | 104060/25 | 39148/10 | 0/0 |
| **Collecting duct tumors** |  |  |  |
| Collecting duct carcinoma | 137119/47 | 46976/21 | 8963/3 |
| **Other renal tumors** |  |  |  |
| Clear cell papillary renal cell tumor | 266581/105 | 64030/28 | 17256/10 |
| Mucinous tubular and spindle cell carcinoma | 195949/60 | 44444/16 | 16140/4 |
| Tubulocystic renal cell carcinoma | 2664/2 | 15795/2 | 5089/3 |
| Acquired cystic disease-associated RCC | 12652/2 | 0/0 | 0/0 |
| Eosinophilic solid and cystic RCC | 4406/2 | 3700/2 | 12925/4 |
| Renal cell carcinoma, NOS | 487511/192 | 197230/60 | 18358/5 |
| **Molecularly defined renal carcinomas** |  |  |  |
| TFE3-rearranged RCC | 412278/166 | 132455/43 | 15683/3 |
| TFEB-altered RCC | 42515/11 | 13584/6 | 24856/7 |
| Fumarate hydratase-deficient RCC | 1923/1 | 15150/5 | 9710/3 |
| HLRCC syndrome-associated RCC | 8910/1 | 462/1 | 2577/1 |
| Medullary carcinoma, NOS | 0/0 | 0/0 | 0/0 |
| Indeterminate pathologic subtypes | 48451/22 | 31085/11 | 3905/1 |
| Normal tissue slide | 716109/388 | 168979/96 | 35699/10 |
| 1. Tumor area here refers to tumor tissues without necrosis or sarcomatoid differentiation. | | | |

Table 4: Sensitivity, specificity and F1 score for tissue detection task

| **Cohort** | **Types** | **Sensitivity (95%CI)** | **Specificity (95%CI)** | **F1 score (95%CI)** |
| --- | --- | --- | --- | --- |
| Internal  validation | Normal | 0.931 (0.931-0.932) | 0.985 (0.985-0.985) | 0.948 (0.948-0.948) |
|  | Tumor | 0.888 (0.887-0.888) | 0.971 (0.977-0.977) | 0.933 (0.933-0.933) |
|  | Necrosis | 0.911 (0.909-0.914) | 0.989 (0.989-0.989) | 0.684 (0.425-0.432) |
|  | Sarcomatoid differentiation | 0.818 (0.813-0.823) | 0.955 (0.955-0.956) | 0.182 (0.180-0.185) |
|  | Pseudocapsule | 0.901 (0.898-0.904) | 0.977 (0.977-0.977) | 0.429 (0.425-0.432) |
| External  test | Normal | 0.938 (0.937-0.938) | 0.995 (0.995-0.995) | 0.963 (0.962-0.963) |
|  | Tumor | 0.945 (0.945-0.946) | 0.946 (0.917-0.922) | 0.958 (0.958-0.958) |
|  | Necrosis | 0.941 (0.937-0.944) | 0.993 (0.993-0.993) | 0.641 (0.637-0.646) |
|  | Sarcomatoid differentiation | 0.509 (0.504-0.514) | 0.979 (0.978-0.979) | 0.350 (0.346-0.353) |
|  | Pseudocapsule | 0.919 (0.917-0.922) | 0.986 (0.986-0.986) | 0.650 (0.647-0.653) |
| 1, Calculate the 95%CI using bootstrap (1000 times) | | | | |

Table 5: Number of image patches and slides analyzed for subtype prediction

| **Variables** | **Multicenter**  **Training set**  **18943469patches**  **/7575slides** | **Multicenter**  **test set**  **4740046patches**  **/1917slides** | **External**  **test set**  **1850296patches**  **/854slides** |
| --- | --- | --- | --- |
| **Tumor regions** (patches/slides) |  |  |  |
| Tumor area^1^ | 16395790/7494 | 4074891/1895 | 1750555/835 |
| Necrosis | 1529920/4965 | 395049/1255 | 40936/583 |
| Sarcomatoid differentiation | 594810/3154 | 175667/796 | 18204/44 |
| Pseudocapsule | 422949/2472 | 94439/603 | 40601/24 |
| Normal tissue | NA | NA | NA |
| **Pathologic subtypes** (patches/slides) |  |  |  |
| **Clear cell renal cell tumors** |  |  |  |
| Clear cell RCC | 14965000/5550 | 3624681/1381 | 1549451/727 |
| **Papillary renal tumors** |  |  |  |
| Papillary RCC | 1133329/670 | 319113/172 | 121878/57 |
| **Oncocytic and chromophobe renal tumors** |  |  |  |
| Oncocytoma | 516061/229 | 114699/50 | 2967/1 |
| Chromophobe RCC | 1263529/556 | 346422/146 | 122564/44 |
| **Collecting duct tumors** |  |  |  |
| Collecting duct carcinoma | 98787/47 | 35755/21 | 5038/3 |
| **Other renal tumors** |  |  |  |
| Clear cell papillary renal cell tumor | 144200/105 | 34904/28 | 9963/10 |
| Mucinous tubular and spindle cell carcinoma | 143750/60 | 29970/16 | 13309/4 |
| Renal cell carcinoma, NOS | 363721/192 | 150624/60 | 11995/5 |
| **Molecularly defined renal carcinomas** |  |  |  |
| TFE3-rearranged RCC | 303585/166 | 83878/43 | 13131/3 |
| 1. Tumor area here refers to tumor tissues without necrosis or sarcomatoid differentiation. | | | |

Table 6: Patch-level sensitivity, specificity and F1 score for classification of nine renal cell carcinoma tasks

| **Cohort** | **Types** | **Sensitivity (95%CI)** | **Specificity (95%CI)** | **F1 score (95%CI)** |
| --- | --- | --- | --- | --- |
| Internal  validation | clear cell renal cell carcinoma | 0.977 (0.977-0.977) | 0.799 (0.798-0.800) | 0.958 (0.958-0.958) |
|  | papillary renal cell carcinoma | 0.807 (0.806-0.808) | 0.989 (0.989-0.989) | 0.823 (0.822-0.824) |
|  | renal oncocytoma | 0.753 (0.750-0.755) | 0.991 (0.991-0.991) | 0.708 (0.706-0.710) |
|  | chromophobe renal cell carcinoma | 0.848 (0.847-0.849) | 0.989 (0.989-0.990) | 0.856 (0.855-0.857) |
|  | collecting duct carcinoma | 0.040 (0.037-0.042) | 0.998 (0.998-0.998) | 0.063 (0.059-0.066) |
|  | clear cell papillary renal cell tumor | 0.433 (0.428-0.438) | 0.998 (0.998-0.998) | 0.508 (0.503-0.513) |
|  | mucinous tubular and spindle cell carcinoma | 0.283 (0.278-0.288) | 0.999 (0.999-0.999) | 0.391 (0.386-0.397) |
|  | renal cell carcinoma, not otherwise specified (NOS) | 0.339 (0.337-0.342) | 0.992 (0.991-0.992) | 0.425 (0.422-0.428) |
|  | TFE3-rearranged renal cell carcinomas | 0.376 (0.373-0.379) | 0.993 (0.993-0.993) | 0.430 (0.427-0.434) |
| External  test | clear cell renal cell carcinoma | 0.990 (0.990-0.990) | 0.625 (0.623-0.627) | 0.960 (0.960-0.960) |
|  | papillary renal cell carcinoma | 0.571 (0.568-0.574) | 0.996 (0.996-0.996) | 0.699 (0.697-0.702) |
|  | renal oncocytoma | 0.004 (0.002-0.007) | 0.998 (0.998-0.998) | 0.004 (0.002-0.006) |
|  | chromophobe renal cell carcinoma | 0.696 (0.693-0.698) | 0.997 (0.997-0.997) | 0.803 (0.801-0.805) |
|  | collecting duct carcinoma | 0.224 (0.212-0.236) | 0.994 (0.994-0.994) | 0.129 (0.122-0.136) |
|  | clear cell papillary renal cell tumor | 0.073 (0.068-0.078) | 0.999 (0.999-0.999) | 0.112 (0.105-0.119) |
|  | mucinous tubular and spindle cell carcinoma | 0.312 (0.304-0.320) | 0.998 (0.998-0.9908) | 0.381 (0.373-0.389) |
|  | renal cell carcinoma, not otherwise specified (NOS) | 0.026 (0.023-0.029) | 0.997 (0.996-0.997) | 0.034 (0.030-0.038) |
|  | TFE3-rearranged renal cell carcinomas | 0.002 (0.001-0.002) | 0.998 (0.998-0.998) | 0.003 (0.002-0.004) |
| 1, Calculate the 95%CI using bootstrap (1000 times) | | | | |

Table 7: Slide-level sensitivity, specificity and F1 score for classification of nine renal cell carcinoma tasks

| **Cohort** | **Type** | **Patients/slides** | **Sensitivity (95% CI)** | **Specificity (95% CI)** | **PPV (95% CI)** | **NPV (95% CI)** | **F1 score (95% CI)** | **PR AUC (95% CI)** |
| --- | --- | --- | --- | --- | --- | --- | --- | --- |
| Internal validation | Clear cell RCC (ccRCC) | 931/1381 | 0.975 (0.966-0.982) | 0.937 (0.916-0.957) | 0.975 (0.968-0.983) | 0.935 (0.915-0.953) | 0.975 (0.969-0.980) | 0.996 (0.993-0.998) |
|  | Papillary RCC (pRCC) | 122/172 | 0.884 (0.837-0.930) | 0.985 (0.979-0.990) | 0.854 (0.808-0.899) | 0.988 (0.984-0.993) | 0.869 (0.833-0.902) | 0.955 (0.935-0.974) |
|  | Renal oncocytoma | 16/50 | 0.840 (0.740-0.940) | 0.991 (0.987-0.995) | 0.724 (0.629-0.824) | 0.996 (0.993-0.998) | 0.778 (0.700-0.849) | 0.818 (0.723-0.897) |
|  | Chromophobe RCC (chRCC) | 96/146 | 0.932 (0.890-0.966) | 0.994 (0.990-0.997) | 0.932 (0.890-0.966) | 0.994 (0.991-0.997) | 0.932 (0.899-0.959) | 0.961 (0.934-0.982) |
|  | Collecting duct carcinoma | 3/21 | 0.095 (0.000-0.238) | 0.997 (0.995-0.999) | 0.286 (0.000-0.667) | 0.990 (0.989-0.992) | 0.143 (0.000-0.323) | 0.312 (0.187-0.511) |
|  | Clear cell papillary renal cell tumor | 11/28 | 0.857 (0.714-0.964) | 0.998 (0.996-1.000) | 0.889 (0.778-1.000) | 0.998 (0.996-0.999) | 0.873 (0.769-0.963) | 0.898 (0.807-0.971) |
|  | Mucinous tubular and spindle cell carcinoma | 5/16 | 0.438 (0.188-0.688) | 1.000 (1.000-1.000) | 1.000 (1.000-1.000) | 0.995 (0.993-0.997) | 0.609 (0.316-0.815) | 0.838 (0.680-0.962) |
|  | RCC, not otherwise specified (NOS) | 14/60 | 0.533 (0.400-0.667) | 0.982 (0.975-0.988) | 0.485 (0.383-0.600) | 0.985 (0.981-0.989) | 0.508 (0.403-0.609) | 0.554 (0.445-0.672) |
|  | TFE3-rearranged RCC | 12/43 | 0.791 (0.651-0.907) | 0.993 (0.989-0.996) | 0.708 (0.600-0.829) | 0.995 (0.992-0.998) | 0.747 (0.652-0.841) | 0.609 (0.488-0.771) |
| External test | Clear cell RCC (ccRCC) | 726/727 | 0.992 (0.985-0.997) | 0.709 (0.630-0.780) | 0.951 (0.939-0.963) | 0.938 (0.889-0.980) | 0.971 (0.964-0.978) | 0.991 (0.985-0.995) |
|  | Papillary RCC (pRCC) | 57/57 | 0.649 (0.526-0.772) | 0.996 (0.991-1.000) | 0.925 (0.841-1.000) | 0.975 (0.967-0.984) | 0.763 (0.659-0.854) | 0.819 (0.733-0.904) |
|  | Renal oncocytoma | 1/1 | 0.000 (0.000-0.000) | 0.999 (0.995-1.000) | 0.000 (0.000-0.000) | 0.999 (0.999-0.999) | 0.000 (0.000-0.000) | 0.056 (0.038-0.100) |
|  | Chromophobe RCC (chRCC) | 44/44 | 0.795 (0.682-0.909) | 1.000 (1.000-1.000) | 1.000 (1.000-1.000) | 0.989 (0.983-0.995) | 0.886 (0.811-0.952) | 0.946 (0.895-0.985) |
|  | Collecting duct carcinoma | 3/3 | 0.333 (0.000-1.000) | 0.992 (0.986-0.998) | 0.125 (0.000-0.376) | 0.998 (0.996-1.000) | 0.182 (0.000-0.500) | 0.456 (0.132-1.000) |
|  | Clear cell papillary renal cell tumor | 6/10 | 0.100 (0.000-0.300) | 0.996 (0.992-1.000) | 0.250 (0.000-1.000) | 0.989 (0.988-0.992) | 0.143 (0.000-0.421) | 0.263 (0.105-0.629) |
|  | Mucinous tubular and spindle cell carcinoma | 3/4 | 0.250 (0.000-0.750) | 0.999 (0.996-1.000) | 0.500 (0.000-1.000) | 0.996 (0.995-0.999) | 0.333 (0.000-0.750) | 0.510 (0.216-0.900) |
|  | RCC, not otherwise specified (NOS) | 4/5 | 0.200 (0.000-0.600) | 0.995 (0.991-0.999) | 0.200 (0.000-0.750) | 0.995 (0.994-0.998) | 0.200 (0.000-0.571) | 0.094 (0.040-0.333) |
|  | TFE3-rearranged RCC | 3/3 | 0.000 (0.000-0.000) | 0.999 (0.996-1.000) | 0.000 (0.000-0.000) | 0.996 (0.996-0.996) | 0.000 (0.000-0.000) | 0.044 (0.030-0.105) |
| 1. Calculate the 95%CI using bootstrap (1000 times) 2. | | | | | | | | |

Table 8: Number of patients and slides analyzed for WHO/ISUP grades prediction

| **Variables** | **Multicenter**  **Training set**  **800 patients**  **/1468 slides** | **Multicenter**  **test set**  **200 patients**  **/372 slides** |
| --- | --- | --- |
| **Clear cell renal cell carcinoma (patients/slides)** |  |  |
| Grade 1 | 33/62 | 6/11 |
| Grade 2 | 456/879 | 108/206 |
| Grade 3 | 75/144 | 19/36 |
| Grade 4 | 36/66 | 10/19 |
| **Papillary renal cell carcinoma (patients/slides)** |  |  |
| Grade 1 | 6/9 | 2/4 |
| Grade 2 | 140/223 | 39/69 |
| Grade 3 | 43/68 | 14/23 |
| Grade 4 | 11/17 | 2/4 |
|  | | |

Table 9: Sensitivity, specificity and F1 score for nuclear grading task

| **Cohort** | **Accuracy** | **Sensitivity (95%CI)** | **Specificity (95%CI)** | **F1 score (95%CI)** |
| --- | --- | --- | --- | --- |
| Test set | 0.864 (0.815-0.910) | 0.708 (0.578-0.833) | 0.909 (0.863-0.952) | 0.699 (0.592-0.800) |
| Internal validation | 0.775 (0.750-0.798) | 0.521 (0.465-0.575) | 0.856 (0.834-0.878) | 0.528 (0.477-0.576) |
| External test | 0.739 (0.707-0.767) | 0.447 (0.383-0.518) | 0.833 (0.802-0.860) | 0.453 (0.393-0.517) |
| 1, Calculate the 95%CI using bootstrap (1000 times)  2, The Internal validation cohort and external test cohort apply the trained model to the entire validation and test cohort to verify performance | | | | |

**Table 10: Clinicopathological information of patients with follow-up information across different cohorts**

| **Variables** | **Multicenter**  **Training set**  **N=3490** | **Multicenter**  **test set**  **N=1209** | **External**  **test set**  **N=660** |
| --- | --- | --- | --- |
| **Median age** (IQR) | 57 (65-48) | 58 (66-49) | 61 (69-53) |
| **Male sex** (%) | 2371 (68.0%) | 829 (68.5%) | 446 (67.6%) |
| **Surgical approach** (%) |  |  |  |
| Radical | 2345 (67.2%) | 719 (59.5%) | 426 (64.5%) |
| Partial | 1108 (31.8%) | 435 (35.9%) | 234 (35.5%) |
| **Pathologic tumor size** (%) |  |  |  |
| Median (IQR) | 4 (5.5-2.5) | 3.5 (5.35-2.5) | 3 (5-2.5) |
| ≤4 | 2010 (57.6%) | 710 (58.8%) | 455 (69.0%) |
| >4 to≤7 | 973 (27.9%) | 287 (23.7%) | 145 (22.0%) |
| >7 to≤10 | 321 (9.2%) | 99 (8.2%) | 40 (6.1%) |
| >10 | 146 (4.2%) | 59 (4.9%) | 19 (2.9%) |
| **TNM stage** (%) |  |  |  |
| I | 2618 (75.0%) | 923 (76.4%) | 536 (81.2%) |
| II | 279 (8.0%) | 96 (7.9%) | 29 (4.4%) |
| III | 410 (11.8%) | 133 (11.0%) | 87 (13.2%) |
| IV | 183 (5.2%) | 57 (4.7%) | 7 (1.1%) |
| **WHO/ISUP Grade** (%) |  |  |  |
| 1 | 247 (7.1%) | 86 (7.1%) | 127 (19.2%) |
| 2 | 2394 (68.6%) | 827 (68.4%) | 376 (57.0%) |
| 3 | 633 (18.14%) | 217 (17.9%) | 125 (19.0%) |
| 4 | 211 (6.0%) | 71 (6.0%) | 31 (4.7%) |
| **Necrosis** (%) | 532 (15.3%) | 161 (13.3%) | 48 (7.3%) |
| **Sarcomatoid differentiation** (%) | 147 (4.2%) | 48 (4.0%) | 16 (2.4%) |
| **Medical centers** |  |  |  |
| Zhongshan Hospital | 3050 | 958 | NA |
| Xiamen clinical cancer center | 24 | 24 | NA |
| Zhangye People’s hospital | 71 | 19 | NA |
| CPTAC-CCRCC cohort | 31 | 47 | NA |
| Huadong Hospital | NA | NA | 660 |
| TCGA cohorts (KIRC. KIRP, KICH) | 314 | 161 | NA |
| **Pathologic subtypes** |  |  |  |
| **Clear cell renal cell tumors** |  |  |  |
| Clear cell RCC | 2830 | 922 | 559 |
| Multilocular cystic renal neoplasm of low malignant potential | 35 | 13 | 6 |
| **Papillary renal tumors** |  |  |  |
| Papillary RCC | 239 | 120 | 38 |
| **Oncocytic and chromophobe renal tumors** |  |  |  |
| Oncocytoma | 0 | 0 | 1 |
| Chromophobe RCC | 244 | 96 | 34 |
| Other oncocytic tumors of the kidney | 4 | 3 | 0 |
| **Collecting duct tumors** |  |  |  |
| Collecting duct carcinoma | 12 | 3 | 2 |
| **Other renal tumors** |  |  |  |
| Clear cell papillary renal cell tumor | 27 | 11 | 6 |
| Mucinous tubular and spindle cell carcinoma | 8 | 5 | 0 |
| Tubulocystic renal cell carcinoma | 1 | 1 | 1 |
| Acquired cystic disease-associated RCC | 1 | 0 | 0 |
| Eosinophilic solid and cystic RCC | 0 | 1 | 3 |
| Renal cell carcinoma, NOS | 44 | 14 | 4 |
| **Molecularly defined renal carcinomas** |  |  |  |
| TFE3-rearranged RCC | 33 | 12 | 1 |
| TFEB-altered RCC | 3 | 1 | 1 |
| Fumarate hydratase-deficient RCC | 0 | 1 | 2 |
| HLRCC syndrome-associated RCC | 1 | 1 | 1 |
| Medullary carcinoma, NOS | 0 | 0 | 0 |
| Indeterminate pathologic subtypes | 8 | 5 | 1 |
| Median follow-up time (years) (IQR) | 6.35 (8.63-5.35) | 5.60 (7.57-3.07) | 5.78 (8.72-3.81) |

# Table 11: Univariate analysis and Multivariate analysis of recurrence-free survival in internal validation cohort

| **Variables** | **Univariate analysis** | | |  | **Multivariate analysis** | | |
| --- | --- | --- | --- | --- | --- | --- | --- |
|  | **Hazard**  **Ratio** | **95%CI** | **p-value** |  | **Hazard**  **Ratio** | **95%CI** | **p-value** |
| **TNM stage** |  |  | <0.001 |  |  |  | <0.001 |
| $\text{Ⅱ vs Ⅰ}$ | 5.024 | 3.177-7.943 | <0.001 |  | 3.626 | 2.267-5.800 | <0.001 |
| $\text{Ⅲ vs Ⅰ}$ | 11.209 | 7.733-16.248 | <0.001 |  | 7.452 | 5.068-10.960 | <0.001 |
| $\text{Ⅳ vs Ⅰ}$ | 40.682 | 24.106-68.656 | <0.001 |  | 26.623 | 15.231-46.535 | <0.001 |
| **ISUP grade** |  |  | <0.001 |  |  |  | <0.001 |
| $\text{high}\text{ vs }\text{low}$ | 6.098 | 4.454-8.348 |  |  | 3.084 | 2.135-4.455 | <0.001 |
| **Necrosis** |  |  | <0.001 |  |  |  | 0.234 |
| $\text{present vs absent}$ | 4.968 | 3.574-6.900 |  |  | 1.273 | 0.855-1.896 |  |
| **Sacromatoid differentiation** |  |  | <0.001 |  |  |  | 0.645 |
| $\text{present vs absent}$ | 7.823 | 4.910-12.463 |  |  | 1.133 | 0.667-1.924 |  |
| **WSI-based risk score** |  |  | **<0.001** |  |  |  | **<0.001** |
| $\text{≥}\text{1 vs 0}$ | 6.492 | 4.413-9.549 |  |  | 2.707 | 1.750-4.187 |  |

# Table 12: Univariate analysis and Multivariate analysis of disease-specific survival in internal validation cohort

| **Variables** | **Univariate analysis** | | |  | **Multivariate analysis** | | |
| --- | --- | --- | --- | --- | --- | --- | --- |
|  | **Hazard**  **Ratio** | **95%CI** | **p-value** |  | **Hazard**  **Ratio** | **95%CI** | **p-value** |
| **TNM stage** |  |  | <0.001 |  |  |  | <0.001 |
| $\text{Ⅱ vs Ⅰ}$ | 6.897 | 3.976-11.964 | <0.001 |  | 4.835 | 2.750-8.499 | <0.001 |
| $\text{Ⅲ vs Ⅰ}$ | 14.141 | 8.963-22.311 | <0.001 |  | 8.801 | 5.464-14.177 | <0.001 |
| $\text{Ⅳ vs Ⅰ}$ | 66.467 | 41.956-105.298 | <0.001 |  | 26.447 | 15.923-43.926 | <0.001 |
| **ISUP grade** |  |  | <0.001 |  |  |  | <0.001 |
| $\text{high}\text{ vs }\text{low}$ | 8.300 | 5.877-11.721 |  |  | 2.489 | 1.636-3.787 |  |
| **Necrosis** |  |  | <0.001 |  |  |  | 0.003 |
| $\text{present vs absent}$ | 7.747 | 5.585-10.746 |  |  | 1.804 | 1.215-2.679 |  |
| **Sacromatoid differentiation** |  |  | <0.001 |  |  |  | 0.065 |
| $\text{present vs absent}$ | 12.164 | 8.020-18.450 |  |  | 1.557 | 0.974-2.490 |  |
| **WSI-based risk score** |  |  | **<0.001** |  |  |  | **0.003** |
| $\text{≥}\text{1 vs 0}$ | 9.563 | 6.227-14.686 |  |  | 2.163 | 1.303-3.590 |  |

# Table 13: Univariate analysis and Multivariate analysis of recurrence-free survival in external test cohort

| **Variables** | **Univariate analysis** | | |  | **Multivariate analysis** | | |
| --- | --- | --- | --- | --- | --- | --- | --- |
|  | **Hazard**  **Ratio** | **95%CI** | **p-value** |  | **Hazard**  **Ratio** | **95%CI** | **p-value** |
| **TNM stage** |  |  | <0.001 |  |  |  | <0.001 |
| $\text{Ⅱ vs Ⅰ}$ | 14.792 | 7.443-29.399 | <0.001 |  | 8.502 | 4.132-17.492 | <0.001 |
| $\text{Ⅲ vs Ⅰ}$ | 13.555 | 7.990-22.993 | <0.001 |  | 7.881 | 4.462-13.920 | <0.001 |
| $\text{Ⅳ vs Ⅰ}$ | 32.813 | 9.778-110.121 | <0.001 |  | 12.529 | 3.180-49.365 | <0.001 |
| **ISUP grade** |  |  | <0.001 |  |  |  | 0.006 |
| $\text{high}\text{ vs }\text{low}$ | 5.713 | 3.605-9.054 |  |  | 2.061 | 1.226-3.466 |  |
| **Necrosis** |  |  | <0.001 |  |  |  | 0.082 |
| $\text{present vs absent}$ | 8.010 | 4.866-13.186 |  |  | 1.696 | 0.934-3.077 |  |
| **Sacromatoid differentiation** |  |  | <0.001 |  |  |  | 0.809 |
| $\text{present vs absent}$ | 9.967 | 4.943-20.101 |  |  | 0.898 | 0.376-2.145 |  |
| **WSI-based risk score** |  |  | **<0.001** |  |  |  | **0.003** |
| $\text{≥}\text{1 vs 0}$ | 11.640 | 5.782-23.394 |  |  | 3.102 | 1.466-6.563 |  |

# Table 14: Univariate analysis and Multivariate analysis of disease-specific survival in external test cohort

| **Variables** | **Univariate analysis** | | |  | **Multivariate analysis** | | |
| --- | --- | --- | --- | --- | --- | --- | --- |
|  | **Hazard**  **Ratio** | **95%CI** | **p-value** |  | **Hazard**  **Ratio** | **95%CI** | **p-value** |
| **TNM stage** |  |  | <0.001 |  |  |  | <0.001 |
| $\text{Ⅱ vs Ⅰ}$ | 21.540 | 9.636-48.153 | <0.001 |  | 11.242 | 4.877-25.914 | <0.001 |
| $\text{Ⅲ vs Ⅰ}$ | 21.006 | 10.826-40.760 | <0.001 |  | 10.899 | 5.391-22.037 | <0.001 |
| $\text{Ⅳ vs Ⅰ}$ | 77.594 | 28.918-208.203 | <0.001 |  | 42.038 | 13.810-127.964 | <0.001 |
| **ISUP grade** |  |  | <0.001 |  |  |  | 0.003 |
| $\text{high}\text{ vs }\text{low}$ | 7.907 | 4.676-13.370 |  |  | 2.496 | 1.375-4.533 |  |
| **Necrosis** |  |  | <0.001 |  |  |  | 0.068 |
| $\text{present vs absent}$ | 10.215 | 6.098-17.111 |  |  | 1.774 | 0.960-3.281 |  |
| **Sacromatoid differentiation** |  |  | <0.001 |  |  |  | 0.500 |
| $\text{present vs absent}$ | 12.212 | 5.964-25.005 |  |  | 0.740 | 0.309-1.773 |  |
| **WSI-based risk score** |  |  | **<0.001** |  |  |  | **0.005** |
| $\text{≥}\text{1 vs 0}$ | 16.709 | 7.514-37.154 |  |  | 3.311 | 1.436-7.634 |  |

# Table 15: Cox proportional-hazards regression analysis of the WRS as a continuous variable

| Split | outcome | Total / N | HR per 1-SD  (95% CI) | P | C-index |
| --- | --- | --- | --- | --- | --- |
| Internal alidation | RFS | 1159 / 160 | 2.08 (1.79–2.42) | <0.001 | 0.751 |
| Internal alidation | DSS | 1210 / 147 | 2.45 (2.06–2.90) | <0.001 | 0.780 |
| Internal alidation | OS | 1210 / 203 | 2.02 (1.76–2.31) | <0.001 | 0.738 |
| External test | RFS | 655 / 75 | 2.31 (1.82–2.93) | <0.001 | 0.745 |
| External test | DSS | 660 / 63 | 2.62 (1.99–3.44) | <0.001 | 0.783 |
| External test | OS | 659 / 99 | 1.70 (1.40–2.06) | <0.001 | 0.672 |

# 1. Methods

## 1.1 Study cohorts and data preprocessing

### 1.1.1 Study population

This study included 7,033 patients and a total of 11,135 whole slide images (WSIs), which were subsequently divided into internal and external datasets.

The internal dataset was sourced from three medical centers (Zhongshan Hospital of Fudan University, Xiamen Clinical Research Center for Cancer Therapy, Zhangye People’s Hospital) and two public datasets (CPTAC and TCGA), comprising a total of 6,156 patients (10,239 WSIs). This dataset was divided into an internal training set and an internal validation set, allocated in an 8:2 ratio based on the number of patients within each pathological category. The internal training set included 4,918 patients (8,154 slides), while the external training set included 1,238 patients (2,085 slides).

The external dataset consisted of 877 patients (896 slides), with all data obtained exclusively from Huadong Hospital Affiliated to Fudan University, for evaluating the model efficacy and performance. The details of all cohorts are provided in Additional file 1: Table 1.

### 1.1.2 Patient cohort

We retrospectively collected 8303 WSIs of 4809 patients who received partial or radical nephrectomy with available H&E slides and pathologically proved renal cell tumor in Zhongshan Hospital from 2005 to 2022, 222 WSIs of 141 patients in Xiamen Clinical Research Center for Cancer Therapy from 2017 to 2022, 110 WSIs of 110 patients in Zhangye People’s Hospital from 2008 to 2022, and 896 WSIs of 877 patients in Huadong Hospital from 2010 to 2023. Because papillary adenomas are clinically silent and usually discovered as incidental findings[1], this subtype was excluded from further analyses. Patients with bilateral or familial renal cell tumors, mixed histologic subtypes, or a prior history of nephrectomy were also excluded. For ccRCC, pRCC and chRCC, we collected one or two WSIs for each patient. For other subtypes of renal cell tumors, we collected up to ten WSIs for each patient. Three experienced pathologists in urology (Run Wang, Qi Sun, and Xue Zhang) re-evaluated the slides and pathological reports to re-assign the histologic subtypes, WHO/ISUP grades, and TNM stage of these cases according to the 2022 WHO Classification of Tumors of the Urinary System and Male Genital Organs, the WHO/ISUP grading system, and the 2017 AJCC TNM staging manual, respectively[2, 3]. Cases with suspicious subtypes and insufficient diagnostic information were excluded. The three pathologists also assigned WHO/ISUP grading to non-clear cell or papillary RCC types, which was permitted by international structured reporting protocols, despite that the grading system has not been validated as prognostic parameters for these subtypes[2]. The Cancer Imaging Archive (TCIA) includes TCGA-KIRC, TCGA-KIRP, TCGA-KICH, and CPTAC-CCRCC. Basic clinical information and WSIs was obtained from GDC Data Portal (https://portal.gdc.cancer.gov/). The subtypes and gradings of each WSI were re-assessed by the three pathologists based on original WSIs and pathological reports from the Cancer Digital Slide Archive (https://cancer.digitalslidearchive.org/). WSIs with pathologic subtype with clinical information obtained from the GDC cancer portal were excluded. Recurrence-free survival (RFS) was defined as the time of surgery to the time of local recurrence or distant metastasis. Disease-specific survival (DSS) was calculated from the time of surgery to the time of death from renal cell tumors and overall survival (OS) was calculated from the time of surgery to the time of death from all causes. We followed the declaration of Helsinki and got approved by the ethic committees of Zhongshan Hospital, Xiamen Clinical Research Center for Cancer Therapy, Zhangye People’s Hospital and Huadong Hospital. Written informed consents were waived by the ethic committees.

### 1.1.3 WSIs acquisition and preprocessing

During preprocessing, each whole-slide image was annotated by experienced pathologists to delineate distinct tissue regions, including normal, tumor, pseudocapsules, necrosis, and Sarcomatoid differentiation tissue. These annotations were subsequently used as reference labels for tissue region identification tasks and using Cytomine for data management.

Since the WSIs from different scanners were stored with distinct pyramid resolutions, all slides were uniformly processed at the same physical resolution (MPP = 1 µm per pixel) by selecting the appropriate image pyramid level without further interpolation. Following resampling, each WSI was divided into non-overlapping 224×224 pixel patches extracted from tissue-containing regions. This target resolution (≈10× magnification) was chosen for three reasons: it provides a common denominator across heterogeneous scanner, including centers whose slides were available only at up to ~10× owing to hardware constraints; it balances the preservation of global spatial context and local morphological detail against the information density and computational cost of gigapixel images; and prior work has shown that lower magnifications (≈5–10×) can match or even exceed higher magnifications (20–40×) for whole-slide classification while substantially reducing computation time [4]. A quality control procedure was applied to exclude patches with poor image quality or insufficient histological information, defined as low color saturation (saturation < 10) or low structural complexity (edge density ≤ 10). These thresholds were empirically determined to remove background while retaining diagnostically relevant tissue areas.

Each retained patch was assigned a label according to the pathologist-provided region-level annotations. In cases where multiple annotation labels overlapped, the label corresponding to the highest pathological risk level was retained, reflecting the dominant diagnostic feature within the patch.

## 1.2 Main model architecture

### 1.2.1 Feature extract

To obtain robust and biologically meaningful representations from histopathological images, each retained patch was encoded using a pretrained patch-level encoder derived from the Prov-GigaPath foundation model. Prov-GigaPath is a large-scale pathology foundation model trained on 1.3 billion pathology image tiles from 171,189 whole-slide images collected across diverse cancer types and institutions[5]. It is built upon a Vision Transformer (ViT)-based architecture, enabling the model to effectively capture fine-grained morphological patterns and long-range contextual dependencies within histopathological images.

In this study, we employed its frozen patch encoder to extract 1536-dimensional embeddings for each patch at 1 µm/pixel resolution, ensuring consistent and high-quality feature representations across slides scanned by different devices and centers. These embeddings were subsequently used as inputs for downstream multi-center classification and model training.

### 1.2.2 Aggregation

To aggregate patch-level embeddings extracted from Prov-GigaPath into patient-level representations, we adopted an Attention-based Multiple Instance Learning (ABMIL) framework[6]. This approach enables the model to learn adaptive attention weights for each patch, allowing the network to focus on diagnostically relevant regions within WSIs.

Each slide is represented as a bag composed of N instances, each vector corresponds to a feature extracted by the Prov-GigaPath encoder. The ABMIL model computes an attention score for each instance using a learnable attention module and the attention scores are normalized across all instances. The patient-level feature representation is then obtained via a weighted aggregation, which is subsequently passed to a classification head for patient-level prediction.

## 1.3 Downstream task model

### 1.3.1 Tissue detection

For the task of tissue detection, which focused on identifying five distinct tissue components (normal tissue, tumor tissue, pseudocapsule, necrosis, and sarcomatoid differentiation), each WSI was manually annotated for these five regions by four experienced pathologists, and model performance was primarily evaluated based on patch-level predictive accuracy. The data details used for training and evaluation are all in Table S2. Previous studies have validated the effectiveness of integrating feature extraction with an MLP-based classifier[7].

The tissue detection task takes 1536-dimensional features as input to a three-layer fully connected prediction head, which designed to map the aggregated feature representation into a five-class prediction output. Specifically, it consists of two intermediate hidden layers of size 2,048 with batch normalization and ReLU activation to enhance nonlinear feature transformation and stabilize training. The final linear layer outputs five logits corresponding to the five target categories. The forward propagation of the prediction head can be formulated as follows:

$$h_{1}=\mathrm{ReLU}(\mathrm{BN}_{1}(W_{1}x+b_{1}))$$

(1)

$$h_{2}=\mathrm{ReLU}(\mathrm{BN}_{2}(W_{2}h_{1}+b_{2}))$$

(2)

$$P=\mathrm{Softmax}(W_{3}h_{2}+b_{3})$$

(3)

where $x$ represents 1536-dimensional feature vector, $BN_{i}$ denotes batch normalization, $ReLU(z)=max(0,z)$ is the rectified linear unit activation function, a softmax normalization, defined via ${[Softmax(z)]}_{i}=\frac{e^{z_{i}}}{\sum_{j=1}^{N} z_{j}}$, converts a vector of raw scores into probabilities that sum to 1, and *P* denotes the predicted probability distribution over the five classes.

The network was optimized using stochastic gradient descent (SGD) for 10 epochs with a batch size of 512. The initial learning rate was set to $1\times{10}^{-4}$. Weighted cross-entropy (class weights: 1:1:5:5:10) with class-dependent resampling. All computations were performed using double-precision (float64) data type. The model was optimized using the cross-entropy loss, and the optimal classification threshold for sarcomatoid differentiation tissue was determined by maximizing Youden’s J statistic.

### 1.3.2 Classification of nine renal cell carcinomas

For the pathological subtype classification of renal tumor, only renal tumor subtypes with a sample size exceeding 10 patients were included to ensure statistical robustness. Model performance was primarily evaluated using slide-level predictive accuracy. A total of nine pathological subtypes were analyzed for classification. The numbers of patches, slides, and patients corresponding to each subtype are detailed in Table S4.

For the subtype classification task, the patches of no-tumor regions were excluded during training. During inference, the classifier trained for tissue region detection was first applied to label tissue regions. The subsequent tasks followed the same pipeline. The 1,536-dimensional feature vectors were also fed into a prediction head to generate the final subtype predictions. The structure of the prediction head is consistent with the tissue detection task, but the output is mapped to predictions of 9 subtypes. After training, patch-level predictions within the same slide were aggregated to obtain bag-level predictions. The final bag label was assigned to the class with the highest ratio, and the mean probability of patches belonging to the class with the highest ratio was used as the bag-level probability. The network was optimized using SGD for 10 epochs with a batch size of 512. The initial learning rate was set to $1\times{10}^{-3}$. All computations were performed using double-precision (float64) data type. The model was optimized using the cross-entropy loss, and the optimal classification threshold for nine subtypes was determined by maximizing Youden’s J statistic.

### 1.3.3 Nuclear grading

For the nuclear grading task, we filtered tumor-related patches and grouped them into bags by patient ID. The original four ISUP grades were simplified into a binary task, grades 1 and 2 were grouped as low grade, while grades 3 and 4 were grouped as high grade.

In this task, we used an ABMIL framework composed of three parts: instance encoder, attention module, and classification head. The effectiveness of this framework has been confirmed in other works[8,9]. The instance encoder consists of two fully connected layers with batch normalization and ReLU activation. The attention module is designed to learn instance-level importance weights within each bag, enabling the model to focus on relevant regions. The attention mechanism computes a normalized attention score for each instance as follows:

$$A_{i}=\frac{\exp(W\tanh(Vx_{i}))}{\sum_{j=1}^{N} \exp(W\tanh(Vx_{j}))}$$

(4)

where *N* denotes the number of instances, *V* and *W* are trainable parameters, tanh introduces non-linearity, $A$ represents the attention score for instance, and the softmax normalization ensures the sum of the attention scores is equal to 1. The aggregated bag-level representation is then obtained by the weighted average of all instance features.

The network was optimized using AdamW for 30 epochs with a batch size of 1. The learning rate was set to $1\times{10}^{-4}$, and a weight decay of $1\times{10}^{-5}$ was applied for regularization. All computations were performed using double-precision (float64) data type. Instead of the standard cross-entropy loss, a multi-class binary cross-entropy loss was employed. This function computes the binary cross-entropy for each class independently and applies class-wise weighting to handle category imbalance more effectively.

### 1.3.4 Prognosis prediction

In this task, instead of using the network for classification, the probability of high-risk categories was used as the risk score as the prognostic risk score. This method has achieved results in other works on renal cell carcinoma[10]. In the training dataset, patients who experienced recurrence during the follow-up period were classified as having a poor prognosis. Conversely, patients who remained recurrence-free throughout the entire follow-up period and had a follow-up duration exceeding 5 years were categorized as having a good prognosis. A total of 3490 patients from the training set exhibited distinct outcomes (either good or poor prognosis), providing a clear ground truth for our analysis. For the prognosis prediction task, the filtering process is consistent with the previous task. We employed the same ABMIL frame as used in nuclear grading task, but reduced the latent feature representation to 512 dimensions. The attention mechanism was further modified by replacing the tanh activation with batch normalization and ReLU functions to enhance feature representation and training stability. The attention mechanism computes a normalized attention score for each instance as follows:

$$A_{i}=\frac{\exp(W_{2}\mathrm{ReLU}(\mathrm{BN}({W_{1}x}_{i}+b_{1})+b_{2}))}{\sum_{j=1}^{N} \exp(W_{2}\mathrm{ReLU}(\mathrm{BN}({W_{1}x}_{j}+b_{1})+b_{2}))}$$

(5)

where *N* denotes the number of instances, $W_{i}$ and $b_{i}$ are trainable parameters, ReLU introduces non-linearity, $A$ represents the attention score for instance, and the softmax normalization ensures the sum of the attention scores is equal to 1. The aggregated bag-level representation is then obtained by the weighted average of all instance features.

The network was optimized using AdamW for 20 epochs with a batch size of 1. The learning rate was set to $1\times{10}^{-4}$, and a weight decay of $1\times{10}^{-5}$ was applied for regularization. All computations were performed using double-precision (float64) data type. The model was optimized using the cross-entropy loss.

## 1.4 Evaluation of downstream tasks

### 1.4.1 Evaluation metrics

For the tissue detection, subtype classification, and nuclear grading tasks, which are multi-class (five-class and nine-subtype) or binary classification problems, model performance was primarily evaluated using the Area Under the Receiver Operating Characteristic curve (AUROC). To provide a more comprehensive assessment, sensitivity, specificity, and F1 score were also calculated for all classification tasks. 95% confidence intervals for all supplementary performance metrics were estimated using a non-parametric bootstrap method[11]. For these threshold-dependent metrics, the optimal classification threshold for each cohort was determined by maximizing Youden’s J statistic. For the prognosis prediction tasks, we further evaluated model performance using Harrell’s concordance index (C-index) to measure how well the predicted risk aligned with the observed event order. Additionally, the time-dependent C-index was employed to assess predictive accuracy at different follow-up time points.

### 1.4.2 Statistical analysis

Statistical analyses were carried out with Python and SPSS Statistics 21.0. Receiver operating characteristic (ROC) curves were performed to assess diagnostic accuracies of the deep learning models. Recurrence-free survival (RFS) was defined as the time of surgery to the time of local recurrence or distant metastasis. Disease-specific survival (DSS) was calculated from the time of surgery to the time of death from renal cell tumors , and overall survival (OS) was calculated from the time of surgery to the time of death from all causes. For patients who did not experience the specified clinical events (recurrence, disease-specific death, or all-cause death) by the end of the follow-up period, or who were lost to follow-up, their data were treated as right-censored at the date of their last clinical contact. The survival curves were plotted using Kaplan-Meier analysis, and statistical significance between risk tiers was evaluated via the two-sided log-rank test. Univariate and multivariable Cox proportional hazards regression analyses were implemented to calculate Hazard Ratios (HRs) and 95% confidence intervals (CIs) to evaluate the independent prognostic value of the WSI-based risk score (WRS). The prognostic discrimination was evaluated using Harrell’s concordance index (C-index). Additionally, to assess predictive accuracy across different longitudinal follow-up time points, time-dependent C-index and time-dependent AUC curves were employed. To account for the dynamic distribution of right-censored patients over time and eliminate potential selection bias, these time-dependent metrics were rigorously computed using the Inverse Probability of Censoring Weighting (IPCW) method. A two-sided p-value < 0.05 was considered statistically significant.

### 1.4.3 Computing hardware and software

Scripts for WSIs pre-processing were written in Python (version 3.10.14) using the libraries Cytomine (version 2.3.0), Numpy (version 2.2.6), Openslide (version 1.4.1), Scikit-image (version 0.25.1), and Pillow (version 10.2.0). All downstream task experiments were conducted on a single 80GB NVIDIA A100 GPU. The experiments were conducted in a software environment consisting of Python 3.10.14, PyTorch 2.3.0, and CUDA 12.1. Specific tasks required additional libraries: Performance metrics were calculated using several Python libraries. Standard classification metrics, including AUROC, PRAUC, sensitivity, specificity, and the F1-score, were computed using Scikit-learn (version 1.7.1). C-index and the time-dependent versions of C-index were calculated using Scikit-survival (version 0.23.1). Implementation of the models was based on the official configurations provided in their respective repositories:

- Prov-GigaPath: https://github.com/prov-gigapath/prov-gigapath
- ABMIL: https://github.com/AMLab-Amsterdam/AttentionDeepMIL

# References:

1.	Grignon DJ, Eble JN: Papillary and metanephric adenomas of the kidney. Semin Diagn Pathol 1998, 15(1):41-53.

2.	Delahunt B, Cheville JC, Martignoni G, Humphrey PA, Magi-Galluzzi C, McKenney J, Egevad L, Algaba F, Moch H, Grignon DJ et al: The International Society of Urological Pathology (ISUP) grading system for renal cell carcinoma and other prognostic parameters. Am J Surg Pathol 2013, 37(10):1490-1504.

3.	Moch H, Amin MB, Berney DM, Comperat EM, Gill AJ, Hartmann A, Menon S, Raspollini MR, Rubin MA, Srigley JR et al: The 2022 World Health Organization Classification of Tumours of the Urinary System and Male Genital Organs-Part A: Renal, Penile, and Testicular Tumours. Eur Urol 2022, 82(5):458-468.

4.	Breen J, Ravikumar N, Allen K, Zucker K, Orsi NM: Reducing Histopathology Slide Magnification Improves the Accuracy and Speed of Ovarian Cancer Subtyping. 2024 IEEE International Symposium on Biomedical Imaging (ISBI) 2024:1-5.

5.	Xu H, Usuyama N, Bagga J, et al: A whole-slide foundation model for digital pathology from real-world data. Nature 2024, 630:181-188.

6.	Ilse M, Tomczak JM, Welling M. Attention-based Deep Multiple Instance Learning. arXiv 2018; published online Feb 13. http://arxiv.org/abs/1802.04712 (preprint).

7.	Courtiol P, Maussion C, Moarii M, Pronier E, Pilcer S, Sefta M, Manceron P, Toldo S, Zaslavskiy M, Le Stang N et al: Deep learning-based classification of mesothelioma improves prediction of patient outcome. Nat Med 2019, 25(10):1519-1525.

8.	Jin D, Liang S, Shmatko A, et al: Teacher-student collaborated multiple instance learning for pan-cancer PDL1 expression prediction from histopathology slides. Nat Commun 2024, 15:3063.

9.	Chen RJ, Lu MY, Williamson DFK, Chen TY, Lipkova J, Noor Z, Shaban M, Shady M, Williams M, Joo B, Mahmood F: Pan-cancer integrative histology-genomic analysis via multimodal deep learning. Cancer Cell 2022, 40(8):865-878.e6.1

10.	Tabibu S, Vinod PK, Jawahar CV: Pan-renal cell carcinoma classification and survival prediction from histopathology images using deep learning. Sci Rep 2019, 9(1):10509.

11.	Chen R, Su Q, Li Y, et al: Multi-sequence MRI-based radiomics model to preoperatively predict the WHO/ISUP grade of clear cell renal cell carcinoma: a two-center study. BMC Cancer 2024, 24:1176.
